# Realising the digital twin: a thematic review and analysis of the ethical, legal, and social issues for digital twins in healthcare

**DOI:** 10.1007/s00146-025-02833-6

**Published:** 2026-02-07

**Authors:** Christopher David Burr, Shuang Qian, Peter Winter, Tim Chico, Camila Rangel Smith, David Wagg, Steven Alexander Niederer

**Affiliations:** 1 The Alan Turing Institute, London, United Kingdom; 2 National Heart and Lung Institute, Imperial College London, London, United Kingdom; 3 University of Bristol, Bristol, United Kingdom; 4 School of Medicine and Population Health, University of Sheffield, Sheffield, United Kingdom; 5 School of Mechanical, Aerospace and Civil Engineering, University of Sheffield, Sheffield, United Kingdom; 6 The Alan Turing Institute, London, United Kingdom; 7 National Heart and Lung Institute, Imperial College London, London, United Kingdom

**Keywords:** Digital twins, Digital healthcare, Data ethics, AI governance, Law, Biomedical ethics

## Abstract

This paper examines ethical, legal, and social issues (ELSI) associated with healthcare digital twins (DTs). Using a systematic thematic analysis, we identify key themes across ethical, legal, and social categories, as well as practical barriers to adoption and implementation. Findings reveal a range of concerns (e.g. lack of empirical validation of DT value propositions) and gaps between the promise of healthcare DTs and the reality of deploying them within existing healthcare systems. By mapping the identified barriers to the Non-adoption, abandonment, scale-up, spread, and sustainability (NASSS) framework, we seek to demonstrate how healthcare DTs require systematic assurance and capability development. We provide practical recommendations for how this can be achieved, with specified stakeholder groups, to help build trustworthy and ethical DTs with demonstrable healthcare value.

**Supplementary Information:**

The online version contains supplementary material available at 10.1007/s00146-025-02833-6.

## Introduction

A 42 year old woman with early-stage breast cancer faces an agonising decision: should she undergo chemotherapy with severe side effects but higher success rates, or choose targeted therapy with fewer side effects but uncertain outcomes for her specific tumour profile? today, this decision relies on population statistics and is characterised by wide-ranging clinical uncertainties. Tomorrow, a digital twin of her tumour, which is continuously updated with data about her treatment responses and real-time biomarkers, could simulate both treatment pathways to help predict which option offers the best outcome—though as this review reveals, significant social, technical, and ethical challenges must be addressed before such personalised simulations become clinical reality.

The allure of the healthcare digital twin (DT) is understandably potent. Imagine a dynamic, virtual replica of yourself, constantly updated with health data, allowing clinicians to predict your response to treatments, simulate surgical outcomes, and tailor preventative strategies with unparalleled accuracy (Coveney 2023). Advances in biomedical science and artificial intelligence (AI) now fuel these computational models, enabling health and healthcare practitioners to synthesise data streams from wearables, electronic health records, genomic sequencing, and medical imaging into actionable, predictive insights.

For the purposes of this review, we define a healthcare digital twin as a virtual information construct that mimics the structure, context, and behaviour of a biological, clinical, or healthcare system, characterised by bidirectional data exchange with its physical counterpart. This definition adapts two existing definitions (Committee et al. 2024; Barn 2022). Healthcare DTs function as sociotechnical systems-of-systems. They integrate multiple modelling approaches—from mechanistic simulations to machine learning—with varied levels of fidelity and synchronisation tailored to specific clinical or organisational objectives.

Proponents position healthcare DTs as enablers of personalised medicine and increased efficiency, addressing a wide variety of healthcare’s uncomfortable realities, such as the apprehension before appointments, diagnostic uncertainty based on population averages, and anxiety from trial-and-error treatments. Yet healthcare DTs face significant challenges. Unlike their engineered counterparts in manufacturing, they model complex, evolving biological systems, introducing inherent complexities in design and development, verification and validation, and reliability.

History reminds us that promising health innovations often fail to bridge the gap between potential and practice due to complex adoption barriers. Why does this promise-reality gap persist?

The non-adoption, abandonment, scale-up, spread, and sustainability (NASSS) framework (Greenhalgh et al. 2017) provides a critical perspective on these failures. The framework identifies seven interrelated dimensions that determine adoption success: the health condition, the technology, its value proposition, adopter capabilities (patients, clinicians, carers), organisational readiness, the systemic context, and embedding and adaptation processes. Technologies falter when encountering misaligned incentives, inadequate training, poor interoperability, algorithmic concerns, or resistance to change (Greenhalgh et al. 2017). Given their complexity, data dependency, and sophisticated algorithms, DTs are particularly vulnerable to NASSS challenges (Winter and Chico 2023). As such, without systemic attention across all seven dimensions, healthcare DTs risk becoming another false promise, failing those who depend on effective healthcare delivery.

This article helps to partially address the challenge of non-adoption for healthcare DTs. The NASSS framework charts structural and operational hurdles, and we build upon its existing normative foundations by highlighting how ethical, legal, and social issues (ELSI) are deeply interwoven with the respective challenges, often acting as root causes or critical modulators of success. These issues are not peripheral checklist items but core components influencing technology use. They shape whether the ‘value proposition’ (dimension 3) appears trustworthy and equitable, determine ‘adopter’ willingness (dimension 4), and align or clash with ‘organisational’ and ‘systemic’ norms (dimensions 5, 6).

We begin with an overview of DT technology (Sect.  2)—readers familiar with computational modelling and digital twin concepts may skip directly to Sect.  3. The methodology section briefly outlines our systematic review approach, with full technical details provided in the Supplementary Material. Our core contribution lies in Sect.  4, presenting eleven major ELSI themes and implementation barriers that all readers should review. Policy-makers and healthcare leaders may particularly focus on our recommendations (Sect.  5), while researchers may find the identified gaps throughout Sect.  4 most valuable for future work. We conclude with a synthesis of key findings and their implications (Sect. 6).

Our interdisciplinary approach bridges technical understanding with ethical considerations, acknowledging both the transformative potential of healthcare DTs and the complex sociotechnical landscape stakeholders must navigate to realise their value. By illuminating these intersections, we offer concrete recommendations to help stakeholders navigate this landscape, fostering innovation that is both powerful and principled.

## Different types of digital twins in healthcare

Healthcare DTs are promised to create virtual representations across a variety of scales, ranging from individual organs to entire healthcare systems. This section groups the myriad implementations into three types: DTs for personalised medicine that enable patient-specific treatment strategies, connected DTs that link virtual patient populations for trial optimisation and population health, and infrastructure DTs modelling hospital dynamics for operational efficiency.^1^ We offer three brief illustrative examples, which highlight various enabling technologies (e.g. hybrid computational models, trusted research environments), and also offer a simple introduction for any readers who may be unfamiliar with key technical concepts or functions.

### Digital twins for personalised medicine

**Fig. 1:**
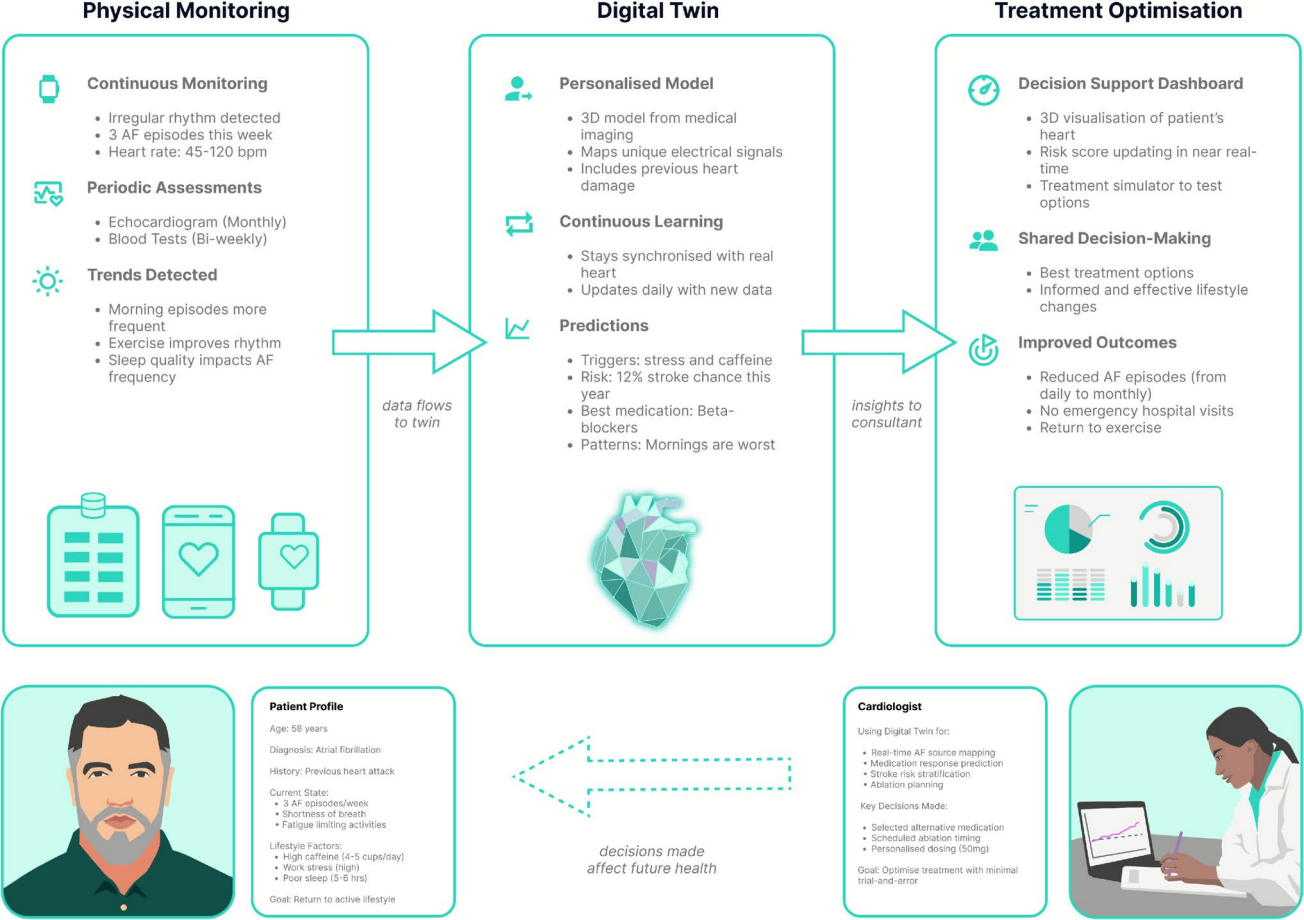
Personalised cardiovascular DT integrating continuous monitoring data to create patient-specific heart models, enabling predictive insights and shared clinical decision-making

DTs for personalised medicine create patient-specific models from organs to whole-body systems, integrating multiple data streams to support clinical decisions about health trajectories and treatment responses. As an example, consider a DT of a patient’s cardiovascular system (Fig. 1)—for instance, a 58 year old patient with atrial fibrillation undergoes continuous monitoring via ECG patches, echocardiograms, and blood tests.

This DT depends on data fusion and integration—a technical challenge of combining heterogeneous data streams that vary in format, frequency, and clinical relevance. Beyond the technical complexity, the DT must temporally align these inputs, standardise different measurement scales, and resolve conflicts between data sources to maintain an ‘accurate’, coherent virtual representation that updates as new information arrives. As we will see, this is not just a technical challenge. Each fusion decision potentially affects clinical outcomes and raises questions about algorithmic transparency and accountability.

This cardiovascular DT employs hybrid modelling. CFD (computational fluid dynamics) models simulate blood flow through patient-specific cardiac geometry. Meanwhile, Machine Learning (ML) algorithms predict arrhythmia onset from ECG patterns and biomarkers. This combination merges mechanistic interpretability with data-driven pattern recognition. Unlike traditional computational modelling, the DT continuously calibrates parameters from incoming data, adjusting predictions as conditions evolve.

Clinical utility of this DT depends on effective data visualisation and dashboard interfaces to support or enhance decision-making. For instance, cardiologists could access interactive 3D heart models with colour-coded abnormalities, overlaid ECG waveforms, and predictive risk scores. These interfaces enable intervention simulation—medication adjustments, ablation modelling, or device therapy prediction.

### Connected digital twins for population health

Connected DTs combine DTs for personalised medicine to create networks of interacting virtual patients. These virtual groups prove valuable for clinical trials, epidemiological modelling, and population health where understanding variability and subgroup responses is crucial.

**Fig. 2:**
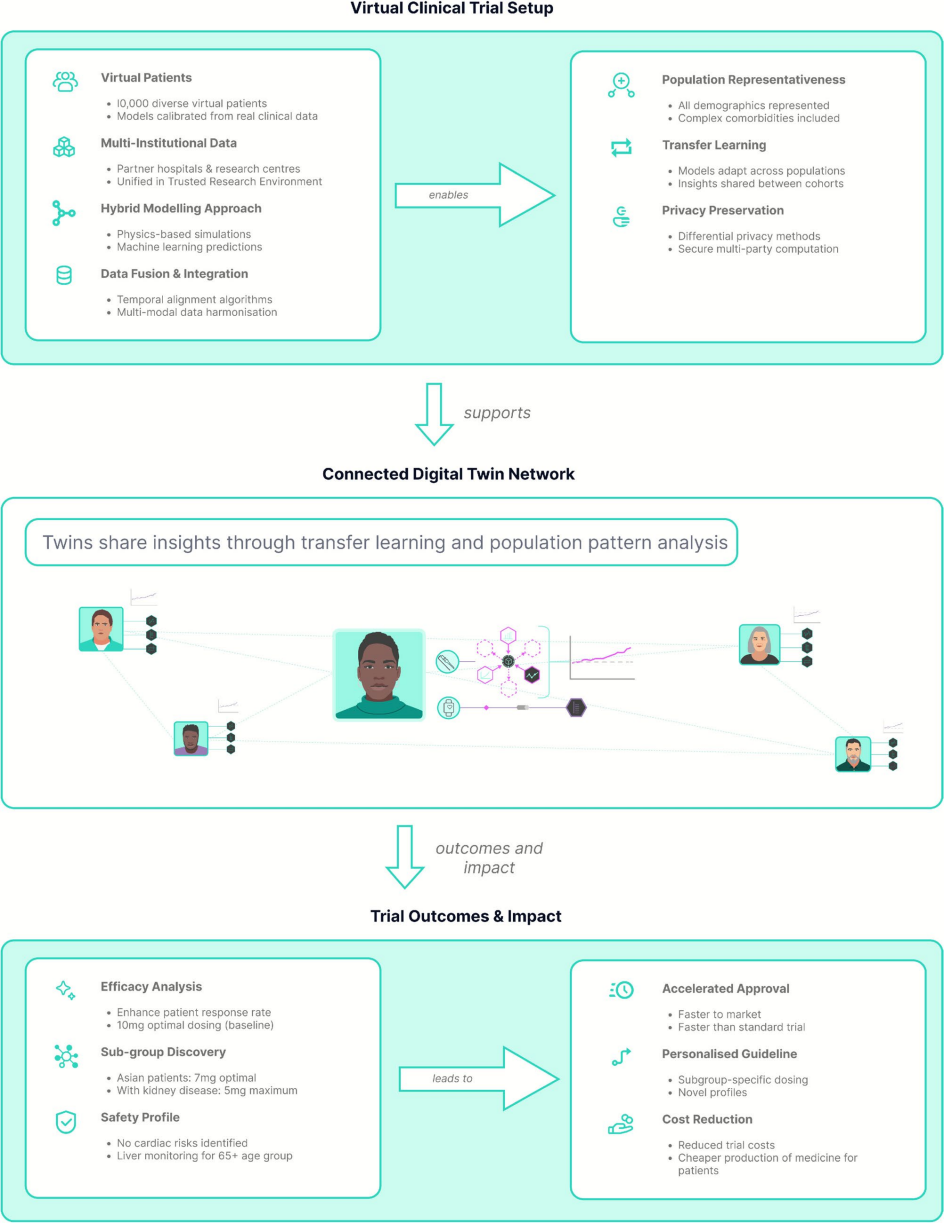
Connected DTs for in silico clinical trials, integrating multi-institutional data within a TRE to create diverse virtual patient populations for early-stage drug development. These computational models align with MHRA’s support for model-informed drug development (MIDD), complementing traditional Phase I-III trials by enabling preliminary efficacy screening and dosing optimisation with privacy-preserving computation and transfer learning capabilities

Consider connected DTs for a diabetes medication trial (Fig. 2). Instead of recruiting thousands of patients for early exploratory studies, 2 this type of DT can create 10,000 +  virtual patients representing diverse populations within a trusted research environment (TRE)—a secure computational infrastructure that supports multi-institutional data analysis while maintaining privacy (Cole et al. 2023). The TRE ingests data from hospitals, universities, and pharmaceutical companies into a unified environment ensuring consistent governance standards and privacy safeguards (Goldacre et al. 2022) 3.

Data integration still employs fusion techniques: temporal alignment for varying sampling frequencies, imputation for missing data, and quality scoring based on reliability. Like the previous type of DT, versioning and provenance documentation ensure appropriate dataset use, creating rich representations of diabetes progression and treatment response. However, connected DTs also enable transfer learning across populations—models trained on one cohort adapt to different demographics. This approach also supports privacy-by-design methods such as differential privacy, secure multi-party computation, and homomorphic encryption.

The increased computational demands of running many simulations drive development of surrogate models—simplified approximations preserving essential dynamics while reducing costs (Kennedy and O’Hagan 2000). These emulators enable real-time treatment exploration.

### Digital twins of healthcare infrastructure

Infrastructure DTs model operational dynamics of physical assets, such as hospitals, or processes such care pathways. They can help optimise resource allocation, predict service bottlenecks, and improve system efficiency (or, ‘strategic planning’) (Croatti et al. 2020).

**Fig. 3:**
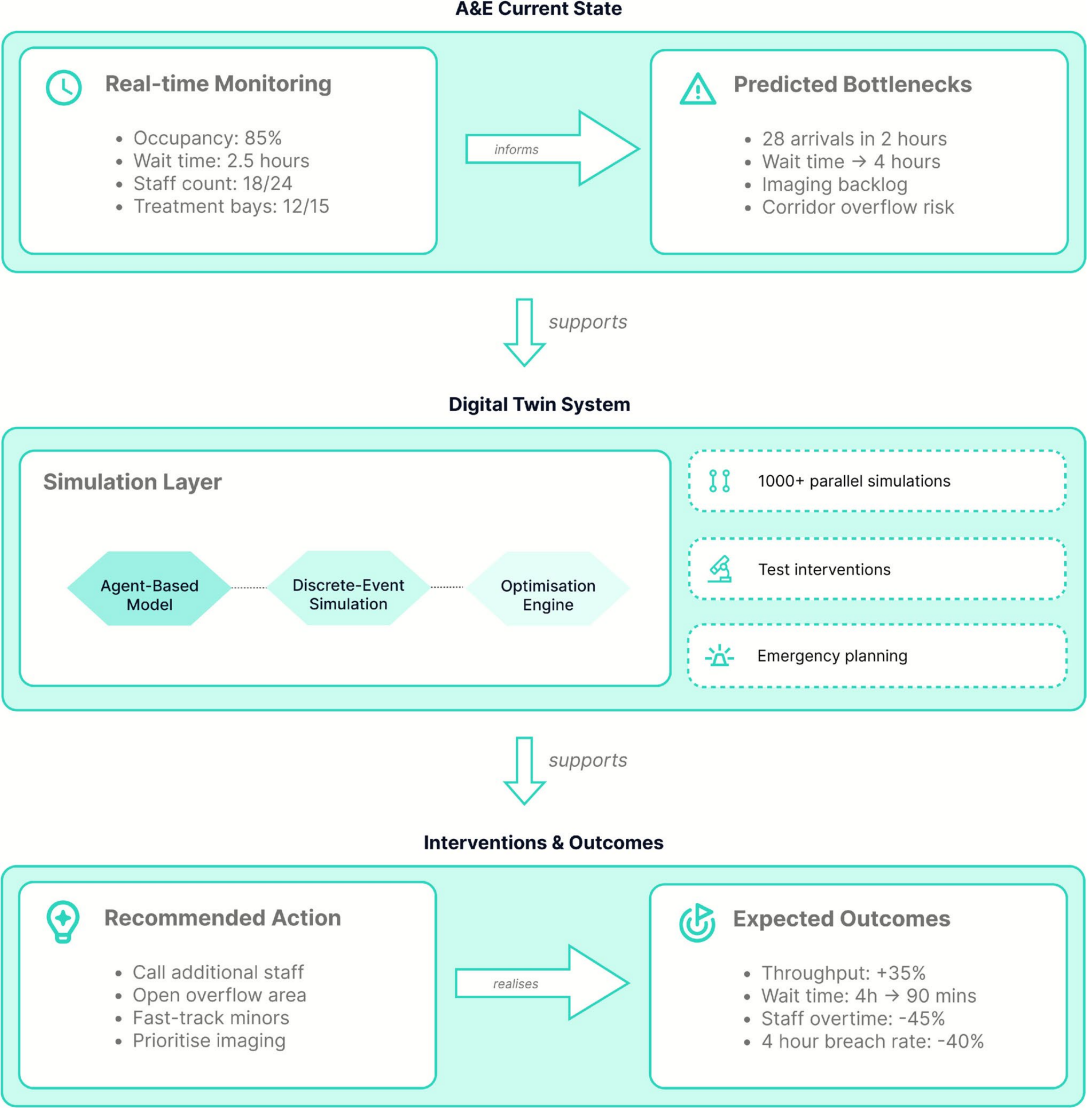
A&E department DT combining agent-based and discrete-event simulation with optimisation algorithms to enhance patient flow, triage prioritisation, and resource allocation. This operational DT provides immediate practical benefits for emergency departments through real-time decision support and capacity planning

Consider an A&E department DT focused on enhancing patient flow and resource utilisation (Fig. 3). The system represents physical spaces, staff, equipment, and patient journeys using discrete-event and agent-based simulation.^4^ In such a system, patients appear as autonomous agents with varying severity ratings and histories; staff function as resource agents with skills and shift patterns; and the environment forms a network of care spaces with capacity constraints.

Infrastructure DTs can support real-time optimisation and decision support through algorithms that dynamically allocate resources, optimise triage decisions, and minimise wait times. For instance, ML models can suggest re-triage of waiting patients based on deterioration risk, while Reinforcement Learning (RL) algorithms balance queue management strategies. Upon detecting bottlenecks, the system generates ranked interventions such as staff reassignment, overflow spaces, or expedited diagnostics, accompanied by predicted outcomes.

Beyond reactive asset and service management, infrastructure DTs could enable scenario planning and stress testing for critical infrastructure resilience. Administrators could simulate emergencies, such as disease outbreaks or equipment failures, to evaluate their preparedness and identify vulnerabilities. By quantifying benefits and unintended consequences before implementation, infrastructure DTs could reduce operational change risks.

While these technical capabilities demonstrate the transformative potential of healthcare DTs, their implementation raises profound ethical, legal, and social questions. To systematically identify and analyse these issues, we conducted a comprehensive thematic review of the emerging literature.

## Review questions and thematic analysis methodology

This section presents a summary of our research questions and methodology for the thematic analysis. Full details are provided in the Supplementary Material.

### Research questions

The following four research questions guided our review and analysis:

- RQ1. Which ethical, legal, and societal issues have been identified and discussed in the existing literature?
- RQ2. How do these issues relate to or intersect with technological developments in the community (e.g. use of AI, physics vs data-driven modelling)?
- RQ3. What gaps, if any, exist in the literature that require further research or development?
- RQ4. Are there current areas where specific stakeholder groups (e.g. policy-makers, developers) could intervene to mitigate specific risks or maximise opportunities?

### Review methodology

We employed thematic analysis (Braun and Clarke 2006) to identify patterns across diverse sources, capturing ethical, legal and social issues from different research communities. We also adopted a mixed-methods approach to understand and measure the (major and minor) theme prevalence (Sect.  4), ensuring our identified themes were statistically grounded and interpretively valid.

#### Overview of key stages

**Fig. 4:**
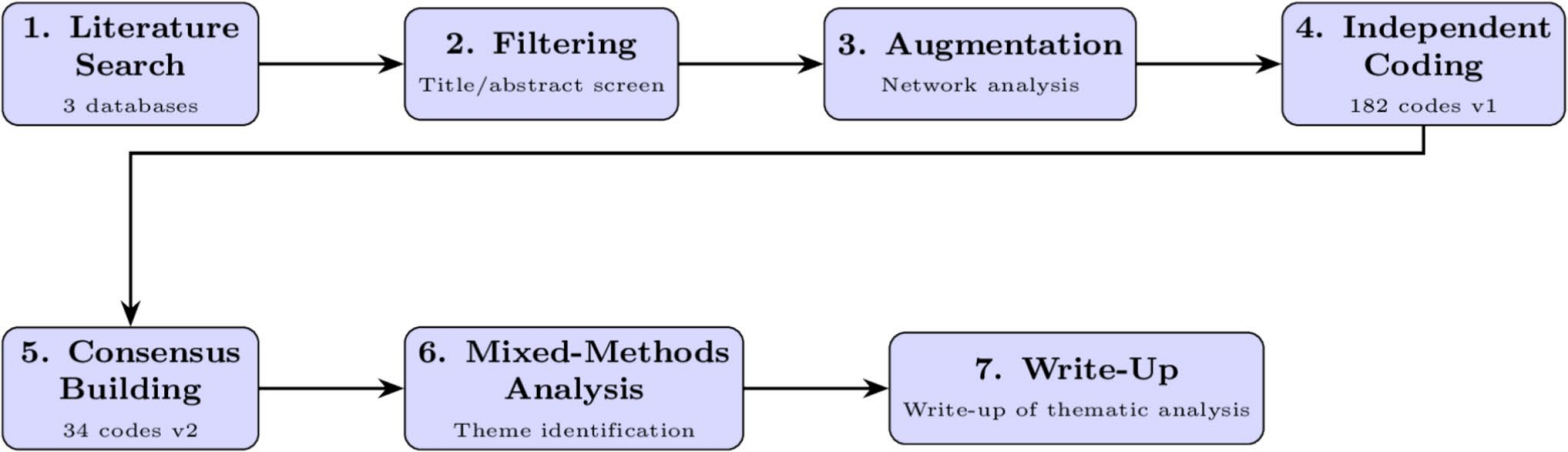
Seven-stage thematic review methodology pipeline

Our review and analysis consisted of the following 7 stages: (1) a systematic search of the published literature; (2) filtering of results; (3) augmentation of preliminary results; (4) independent coding by three reviewers; (5) consensus-building to identify initial themes; (6) mixed-method analysis; (7) validation and write-up (Fig. 4).

The initial search yielded 106 papers from IEEE, PubMed, and ScienceDirect. Title/abstract screening reduced this set to 23. We then used ResearchRabbit 5 to augment the initial set and identify 6 additional papers, resulting in 29 unique papers for analysis.

Three coders independently reviewed these 29 papers, developing 182 codes. Consensus-building refined these to 34 codes across four categories: ethical (10), social (9), legal (6), and barriers (9). Using these codes, we performed additional quantitative analysis to distinguish major/minor themes using weighted composite scoring. This resulted in 11 major themes presented in the next section, with minor themes detailed in the Supplementary Material.

#### Limitations

As with any thematic analysis, there are limitations to our approach that should be acknowledged: selection bias towards technical papers (IEEE, PubMed) under-representing legal issues; scope limited to “digital twins” terminology excluding ELSI discussions in related areas such as AI governance or biomedical ethics; systematic category imbalances suggesting social/legal themes undervalued despite category-specific corrections. A discussion of the full limitations is provided in the ‘Supplementary Material’.

Despite these limitations, our analysis revealed critical insights about the ethical, legal, and social landscape surrounding healthcare DTs. The following section presents our findings, organised into eleven major themes that emerged from the systematic coding process.

## Thematic analysis

Our analysis examines risks and opportunities concerned with ELSI, alongside barriers to realising these opportunities and mitigating the risks. This dual perspective ensures our analysis remains pragmatic and provides a grounded assessment of implementation challenges. In each sub-section we present a summary of the major themes, as well as addressing any gaps noted during our analysis, with minor themes detailed in the Supplementary Material. However, before we turn to the ELSI themes and barriers, we begin by addressing a recurrent and general theme.

### General analysis: the gaps between promises and reality

Our thematic analysis reveals a persistent gap between DT aspirations and present capabilities. The literature often describes DTs as transformative (Mohr et al. 2024), yet overlooks substantial barriers to realising this transformation (e.g. proposing data integration without addressing heterogeneity, touting real-time monitoring without acknowledging infrastructure limitations). Table 1 systematically contrasts promises with realities of current healthcare systems.^6^

**Table 1:** Promise-reality gap analysis in healthcare DTs literature

Domain	Promise	Reality	Gap
Data & integration			
Seamless Integration	Seamless multi-modal data fusion from IoT devices, EHRs, medical imaging	Standardised approaches to data fusion currently lacking; heterogeneous systems, incompatible formats	Large
Equity & access			
Democratised care	DTs enabling universal access to precision medicine across all populations	Digital divide; resource-constrained hospitals lack infrastructure; marginalised communities excluded	Large
Clinical impact			
Personalised treatment	DTs overcoming high medication ineffectiveness rates through individualised solutions	Low empirical evidence for organ-based DT effectiveness; validation approaches undefined	Medium
Predictive Prevention	Long-term disease prediction enabling early intervention	Limited predictive accuracy; risk of overdiagnosis and worried well phenomenon	Large
Economic value			
Cost reduction	Substantial healthcare cost savings and reduced emergency admissions	Evidence from operational twins only; organ-based DTs require high-levels of compute	Medium
Clinical trials	In-silico trials augmenting/replacing human subjects; accelerated drug development	Regulatory uncertainty; fidelity questions; regulatory frameworks still developing	Medium
Infrastructure			
Real-time monitoring	Continuous patient monitoring with instantaneous clinical alerts via IoT/cloud	Intermittent connectivity; limited bandwidth; no operational cloud platforms yet	Large
Trust & Governance			
Privacy protection	Secure, patient-controlled health data ecosystems	Surveillance risks; commercial exploitation; widespread data breaches	Medium
Regulatory approval	Clear pathways for AI-enabled precision medicine	Absence of accepted standards; validation criteria undefined; compliance monitoring gaps	Large

This promise-reality gap reflects deeper epistemological issues about how emerging technologies are conceptualised. Many of the benefits and risks identified in the literature remain speculative rather than empirically validated, raising questions about which claims will materialise. The frequently cited ‘reduced socioeconomic costs’ exemplifies this uncertainty. Benefits from DTs only materialise after achieving critical mass through widespread adoption, but initial development can require potentially prohibitive investments (see Sect. 4.4). In addition, while proponents envision system-wide savings, benefits may accrue primarily to well-resourced organisations who can afford applications like in silico clinical trials, potentially exacerbating rather than alleviating healthcare inequities.

### Ethical issues: themes and gaps

Our analysis identified four major themes dominating ethical discourse: privacy and data governance, equity and accessibility, bias and discrimination, and improved health outcomes. These reflect both traditional bioethical concerns amplified by DT technologies and novel challenges unique to virtualised patient representations.

#### Major themes

##### Privacy and data governance

The tension between data utility and privacy protection emerges as the most prominent ethical (as well as legal) concern, directly affecting the NASSS ‘value proposition’ (dimension 3) by determining whether patients perceive benefits as outweighing privacy risks. DTs require unprecedented data collection—continuous physiological monitoring, genomic information, behavioural patterns, and environmental exposures. Understandably, therefore, the literature reveals particular concern about the potential for data misuse, with (Dahir et al. 2023, p. 82) warning that insurers could use “health-monitoring systems that might motivate a person to exercise and eat well” to “increase rates or exclude so-called high-risk patients.” Such concerns impact ‘adopter’ readiness (dimension 4), as patients may resist participation if they fear discrimination.

These concerns will vary by jurisdiction. While systematic evidence of DT-based discrimination remains limited, precedent exists from genetic testing (Stephens 2025). And, following the Genetic information nondiscrimination act (GINA) in the United States, genetic discrimination proved rare but persistent (Clayton et al. 2019).

However, DTs also present unique data privacy and protection challenges by aggregating continuous, multi-modal data streams that may reveal novel patterns or insights. The EU’s general data protection regulation (GDPR) provides stronger protections through restrictions on automated health data decision-making, yet the global regulatory landscape remains fragmented (Voigt and Bussche 2024). Data governance challenges also multiply with increased data flows to diverse stakeholder groups. Healthcare institutions, technology companies, researchers, and patients all claim legitimate but conflicting interests in DT data.

Related to this, Winter and Chico (2023, p. 12) note how companies “make millions of profit out of patients’ data” while patients “won’t receive any cut and will have to pay to have access to this service.” The literature highlights the struggle to balance individual privacy concerns against collective benefits, and current governance frameworks inadequately manage multi-stakeholder dynamics (Popa et al. 2021; Huang et al. 2022; Bruynseels et al. 2018), necessitating regulatory approaches beyond traditional consent toward dynamic, context-aware mechanisms (Iqbal et al. 2022; Vallée 2024).

This commercial exploitation of health data through DTs exemplifies what Zuboff (2019) terms ‘surveillance capitalism’, which here can be thought of as the extraction of human behavioural and health data for predictive products and services (e.g. health and well-being apps). Healthcare DTs could transform patients into sources of ‘behavioural surplus,’ where continuous physiological monitoring, lifestyle patterns, and treatment responses become raw material for proprietary algorithms (also see (Abad et al. 2021)). Unlike traditional medical records, sufficiently high-fidelity and interconnected DTs could capture the full context of human experience through ambient monitoring. This risks the creation of unprecedented asymmetries of knowledge and power where institutions gain comprehensive insight into individual and population health patterns while patients lose visibility into how their data generates value. Furthermore, the continuous learning capabilities of DTs intensify these dynamics, as each interaction produces more behavioural surplus for algorithmic refinement, creating what Liu et al. (2025) identify as self-perpetuating cycles of data extraction that challenge conventional consent models.

##### Equity, justice, and accessibility

Like any new technology, DTs threaten to create new forms of healthcare stratification between the ‘digitally twinned’ and the ‘digitally excluded.’ Drummond and Coulet (2022) articulate a triple threat to equity: economic barriers preventing disadvantaged families from affording DT services, digital literacy gaps hindering effective use even when access exists, and self-exclusion patterns where marginalised communities opt out of participation due to historical mistrust or cultural factors.

Resource-constrained hospitals in rural regions lack infrastructure for DT implementation, creating geographic inequities mirroring healthcare deserts (Drummond and Coulet 2022; Kamel Boulos and Zhang 2021). Ironically, populations with the greatest health needs will least likely benefit from personalised medicine advances (Bruynseels et al. 2018; Popa et al. 2021). The literature offers few concrete solutions beyond calls for ‘inclusive design,’ leaving tensions between DT aspirations and healthcare justice unresolved (Boretti 2025; Tsekleves et al. 2025).

##### Bias, fairness, and discrimination

While the previous section examined broad systemic inequalities in healthcare DT access, this theme addresses a more specific concern: how algorithmic bias within DT systems themselves perpetuates discrimination. Concerns about algorithmic discrimination are well-studied in the AI fairness literature 7. However, Bruynseels et al. (2018, p. 5) provide a complementary analysis of how DTs could fundamentally alter health conceptualisation, exacerbating algorithmic bias:

“What previously was regarded as healthy, i.e., the absence of any obvious disease indications, can lose its unproblematic character in view of this transparency. Gradations in levels of ‘healthy’ will become pronounced against the backdrop of this data landscape”.

Instead of the traditional binary of ‘healthy’ or ‘sick’, DTs could create hundreds of gradations—individuals classified as ‘87% healthy from a cardiovascular perspective’ or ‘showing early markers for potential metabolic syndrome in 5–7 years’. This granular health stratification fundamentally changes discrimination’s basis. Where discrimination traditionally occurred based on manifest conditions (e.g., denying insurance to someone with diagnosed diabetes), DTs enable discrimination based on probabilistic predictions. These predictive labels affect not just insurance and employment, but also social relationships, as people might avoid romantic partners or friendships based on predicted health trajectories.

These novel forms of discrimination build upon well-documented patterns of algorithmic bias against established social groups in healthcare AI (e.g., class, race, gender) (Liu et al. 2025). Training datasets reflecting historical healthcare disparities, for instance, can lead algorithms to encode and perpetuate inequities (Barocas and Hardt 2023; Obermeyer et al. 2019). Obermeyer et al. (2019) demonstrated that a widely used algorithm systematically underestimated Black patients’ healthcare needs compared to equally sick White patients. For DTs, the continuous ‘digital thread’—used to record continuous data, track clinical decisions, and feed back to update models—could entrench such biases through self-reinforcing cycles. For example, when algorithmic predictions under-serve certain groups and these biased decisions generate outcomes that become subsequent training data, the model learns from its own discriminatory patterns. Unlike static algorithms that can be audited once, continuously learning DTs could progressively amplify biases with each iteration, making discrimination increasingly difficult to detect and reverse.

Additional concerns arise with “self-fulfilling prophecies”. Bruynseels et al. (2018, p. 8) warns that “the mere fact that other people or institutions think that you are going to be sick or weak or short-lived may make you sick, weak or short-lived”—a concern that may be more prominent in mental healthcare or for specific illnesses or health issues (e.g. pain management). This psychosocial dimension extends beyond technical fairness to encompass how DT predictions shape identity, behaviour, and social interactions. The literature reveals deep uncertainty about managing these cascading effects when DTs make extended health predictions, potentially stigmatising individuals based on probabilistic futures rather than present realities.

##### Improved health outcomes

Despite ethical concerns, the literature remains optimistic about DTs revolutionising healthcare through personalisation (see ‘personalised treatment’ in Table 1). Kamel Boulos and Zhang (2021) quantify treatment limitations using 2013 FDA statistics showing medication ineffectiveness rates between 38 and 75% across conditions from depression to cancer, attributed to inter-patient differences that one-size-fits-all approaches cannot address. DTs promise to model “individual patients with varying physiological traits and mechanistic differences,” (Kamel Boulos and Zhang 2021, p. 2) enabling treatments tailored to unique biology, lifestyle, and environment. This extends beyond drug selection to dosing optimisation, intervention timing, and preventive strategies calibrated to individual risk profiles.

DT predictive capabilities promise to shift healthcare from reactive treatment to proactive prevention. Fuller et al. (2020, p. 108,964) highlights systems that “monitor, diagnose and predict the health of a patient” through integrated IoT, cloud, and DT technologies, enabling earlier intervention when treatments prove most effective. Several papers envision DTs detecting disease signatures years before symptoms manifest, altering disease trajectories through timely intervention. However, these predictive capabilities could generate anxiety, overtreatment, and medicalisation of normal variation if not managed carefully. This echoes the well-known critique from Ivan Illich where healthcare systems create the very conditions they claim to cure (Illich 1976). That is, the same predictive power that promises prevention may transform healthy individuals into perpetual patients. The literature’s uncritical embrace of early detection assumes that more information invariably improves outcomes, yet behavioural economics research demonstrates how prediction without actionable intervention can decrease wellbeing through nocebo effects and decision paralysis. The ethical challenge lies in realising benefits while avoiding the ‘worried well’ phenomenon where constant monitoring creates its own pathologies.

Six minor ethical themes—including autonomy and agency, transparency and explainability, and security and surveillance—are discussed in the Supplementary Material (Sect.  3).

#### Gaps

Our analysis revealed two gaps. First, DTs persisting beyond death create new ethical challenges. Unlike static digital assets, healthcare DTs can continue learning posthumously from population data, raising questions about identity, consent, and posthumous autonomy that existing bioethical frameworks cannot address. To better understand this gap, imagine the following hypothetical question and then imagine testing the hypothesis using a patient’s DT: ‘Was there anything we could have done for this patient to prevent them from dying?’ the literature’s focus on living patients ignores that DTs may outlive physical counterparts, potentially serving research or clinical decision-making decades after death without clear ethical guidelines.

Second, population-level DTs for community health and pandemic modelling create unprecedented challenges for collective consent that individual-focused ethical frameworks cannot resolve. When entire populations contribute data to communal DTs that inform clinical trials or public health decisions (see Sect.  2), traditional consent models collapse. However, these same systems may benefit population health tremendously while potentially disadvantaging specific subgroups through algorithmic recommendations that amount to ‘algorithmic gerrymandering’.

The literature’s emphasis on individual (or agent-focused) ethics fails to address how DTs operating at population scale fundamentally challenge liberal bioethical principles based on individual autonomy, creating urgent need for frameworks that can balance collective benefit against individual rights in ways current scholarship has not yet contemplated. Notably, this challenge of balancing collective benefit against individual rights in algorithmic systems is well-explored in the broader data and AI ethics literature, where concepts such as algorithmic accountability, fairness-aware machine learning, and democratic AI governance provide established frameworks that could be adapted for DT applications (Barocas and Hardt 2023). The convergence of DT and AI ethics suggests opportunities for cross-pollination rather than developing entirely novel ethical frameworks.

### Legal issues: themes and gaps

Healthcare DT legal frameworks remain nascent and fragmented due to jurisdictional complexity and tensions between enabling innovation and ensuring protection. Our analysis reveals three major themes: data protection and privacy regulations, regulatory approval and governance mechanisms, and intellectual property and ownership rights. These highlight challenges of applying existing frameworks to virtualised patient representations and complex architectures blurring boundaries between patient data, medical devices, and medical practice.

#### Major themes

##### Data protection and privacy

8Building on the ethical privacy concerns discussed earlier, the legal dimension focuses on regulatory compliance challenges. Bruynseels et al. (2018, p. 8) note how “privacy concerns raised in genomics will be even more relevant in DTs, since the combination of multiple layers of biological and behavioral data will be much more telling about a person than genomics data alone”. This layering effect challenges consent mechanisms, data minimisation, and purpose limitation—core GDPR tenets assuming bounded collection (cf. Table 1). DT data defies traditional legal categories, existing simultaneously as medical records, research data, and real-time monitoring.

In addition, the literature insufficiently addresses the shift from static data collection to continuous IoT and ambient monitoring streams—a fundamental change challenging privacy frameworks designed for episodic encounters—and the related power asymmetries for institutions that hold dynamic, comprehensive digital profiles noted by Popa et al. (2021).

##### Regulatory approval and governance

Regulatory complexity spans pre-market approval and ongoing compliance. Coorey et al. (2022, p. 8) note “regulatory and legal issues for a health digital twin are as yet uncertain but are likely to be especially demanding for approval of devices associated with medical cyber-physical systems that contain large amounts of embedded software”. Compliance monitoring requires “real-time auditing of test results and data access” (Mohr et al. 2024, p. 14), yet traditional post-market surveillance cannot accommodate continuously learning systems.

The regulatory landscape remains uncertain as agencies grapple with evaluating technologies that blur boundaries between medical devices, software, and clinical decision support. Leo et al. (2022, p. 7) note that, “While current regulations do not allow simulations to support FDA approval and European CE marking, both the FDA and EMA are working on this issue.” This creates an impasse: DTs need regulatory approval for clinical adoption, yet regulators lack frameworks for evaluating continuously learning, patient-specific models.

However, this regulatory gap has begun to narrow since 2022, with the UK medicines and health regulatory agency (MHRA), US food and drug administration (FDA), and health Canada publishing guidance such as the ‘predetermined change control plans for machine learning-enabled medical devices’ or ‘software and AI as a medical device (SaMD)’ guidance (Medicines and Healthcare products Regulatory Agency 2023, 2025). More work will be needed to ensure sufficient assurance for the continuous learning, patient-specific models that characterise healthcare DTs. The European medicines agency has initiated discussions on regulatory science frameworks for in silico models, but comprehensive guidelines specifically for healthcare DTs remain in development (European Medicines Agency 2024).

##### Intellectual property and ownership

DTs challenge fundamental assumptions about health data ownership, creating complex webs of intellectual property claims that current legal frameworks struggle to untangle. Zhang et al. (2024, p. 6) articulate a paradigm shift: “Currently, consent for data provision is received from the data producers, which has led to a digital economy built on centralized data owned by large tech corporations. Where the data creators (the patients or participants in research) have limited control and oversight of downstream processes.” This concentration of ownership rights in institutional hands raises vital legal (and social) questions about equitable ownership, particularly when patient data generates significant commercial value through DT development while patients receive no economic benefit.

The ownership complexity multiplies when considering the various components of a DT system—raw patient data, processed datasets, algorithms, model parameters, predictions, and insights all potentially carry different ownership claims. Kamel Boulos and Zhang (2021, p. 9) highlight the governance challenge: “in practice, control will also be shared (and often managed) by the clinical or public health organisation involved in developing or using these DT representations, hence, again, the need to have robust governance mechanisms”.

Overall, the literature reveals deep uncertainty about balancing individual rights with institutional interests, particularly when DTs generate valuable intellectual property through the synthesis of multiple patients’ data. Several papers point to novel ownership models including data trusts, patient cooperatives, or benefit-sharing arrangements (Zhang et al. 2024; Kamel Boulos and Zhang 2021; Huang et al. 2022). However, legal precedents remain undeveloped, leaving ownership questions largely unresolved in practice.

Three minor legal themes—human rights and legal status, liability and accountability frameworks, and contractual and cross-border issues—are analysed in the Supplementary Material (Sect.  3).

#### Gaps

The legal framework gap for healthcare DTs reveals a fundamental mismatch between rapidly evolving technology capabilities and regulatory/governance infrastructure designed for simpler medical interventions. Cross-border data governance presents a particularly thorny concern. For example, the EU has not granted China an adequacy decision under GDPR (Commission 2025), while China’s Personal Information Protection Law (PIPL) emphasises data localisation requirements particularly for large amounts of sensitive personal information (Li et al. 2023), creating compliance challenges where multinational healthcare platforms must maintain conflicting data governance frameworks.^9^ This effectively fragments DT capabilities along national boundaries rather than enabling the global health collaboration these technologies promise.

Liability attribution also represents an equally fundamental gap where current medical malpractice frameworks prove entirely inadequate for DT decisions, especially where opaque forms of machine learning or artificial intelligence are involved. Unlike traditional medical devices with clear manufacturer responsibility, recommendations by AI-enabled DTs emerge from interactions between developers, healthcare providers, algorithm creators, data suppliers, and continuous learning systems that blur traditional causation chains. When DT guidance leads to patient harm, existing legal frameworks may struggle to determine whether fault lies with the physician who followed algorithmic recommendations, the developers who created the model architecture, the institutions that provided training data, or the patients whose behaviour patterns influenced model updates. This can lead to a legal accountability vacuum that undermines both patient protection and provider confidence in DT adoption.^10^

### Social issues: themes and gaps

Social implications of healthcare DTs extend beyond individual care to reshape healthcare systems, professional practices, and societal relationships with health. The initial 9 themes proved challenging to split into major/minor themes (see ‘Supplementary Material’). We therefore grouped 8 themes into four clusters: access and equity (‘social equity & accessibility’ and ‘economic impacts & value proposition’), system transformation (‘healthcare system transformation’ and ‘care delivery & patient experience’), trust and acceptance (‘public trust & social license’ and ‘surveillance, stigma & social control’), and knowledge advancement (‘public health advancement & public literacy’ and ‘evolution of societal norms’).

#### Thematic analysis

##### Access and equity

DTs embody a fundamental healthcare equity dilemma. Kamel Boulos and Zhang (2021, p. 9) claim that DTs “can be seen as a ‘social equaliser’” delivering benefits through “better precision public health interventions”, yet “might not be accessible for every individual or community”. This digital divide arises through many interacting barriers and systemic inequities (e.g. socioeconomic status, technological literacy, geography) where institutions serving disadvantaged populations lack DT resources. The digital divide also reflects broader health equity challenges where new technologies benefit advantaged populations first (Braveman and Gottlieb 2014).

Economic dimensions compound this digital divide. Chase et al. (2023, p. 458) notes healthcare grows at 5–9% annually, consuming 10–18% of GDP, outpacing GDP growth where ability to provide care is outpaced by demand. DTs require substantial infrastructure investments, potentially channelling limited funds toward technology rather than basic access. While evidence shows enthusiasm for cost reduction—“900 percent cost savings” and “61 percent reduction in blue code hospital events” (Rahman et al., cited in Winter and Chico 2023, p. 14)—these derive from hospital operational DTs, not patient-specific applications. This economic framing reveals a troubling pattern: the literature celebrates efficiency gains while overlooking opportunity costs. Investment in DT infrastructure may deepen the ‘inverse care law’ where those with greatest health needs receive least care (Hart 1971). The focus on operational efficiency metrics obscures distributional questions about who benefits from these technologies and whether DT investments address fundamental health inequities or merely optimise care for populations already well-served by existing systems.

##### System transformation

Healthcare DTs are reshaping not merely clinical practices but the fundamental epistemology of medicine and patient care. Lupton (2021, p. 409) emphasises the significance of conceptual framing: “Language is important. It matters. This choice of metaphor can operate persuasively to influence decisions about healthcare funding and practice, excluding alternatives that may be more effective and ethical.” Although the physical twin (i.e. the human patient) is implicitly referenced, the DT metaphor privileges computational simulation over embodied human experience, potentially altering how healthcare professionals conceptualise patients and therapeutic relationships. This (further) shift toward virtualised care delivery raises important questions about the nature of medical practice and patient-provider relationships, which many countries are still grappling with following the COVID-19 pandemic.

The transformation extends to research and development paradigms, with Croatti et al. (2020, p. 3) describing the vision of “making mistakes on computer models instead of people.” While the promise of safer drug development and treatment optimisation cannot (and should not) be ignored, it also represents a fundamental shift in medical epistemology—from learning through direct patient interaction toward knowledge generation through computational simulation (e.g. in silico trials). The literature suggests this transformation may improve treatment precision while potentially diminishing clinical skills that depend on direct patient observation and experiential learning. For instance, Iqbal et al. (2022, p. 593) warns of “digital captivity” where “healthcare providers. may be drawn to” DT models “in a way that they make it their primary treatment object,” potentially displacing attention from actual patients to their virtual representations.

##### Trust and acceptance cluster

Public trust in healthcare DTs emerges as a critical social license concern,^11^ complicated by broader anxieties about artificial intelligence, data surveillance, and technological dependence in healthcare. Lauer-Schmaltz et al. (2024, p. 86,954) identify how systemic risks, such as “an over-reliance on [human DTs], especially in healthcare, might lead to mistrust in professionals in favor of AI-driven decision-making, which can be unreliable due to data biases and inaccuracies.” This dynamic creates potential for trust displacement. Patients may develop greater confidence in algorithmic predictions than clinical expertise, while simultaneously healthcare professionals may become dependent on DT insights, potentially eroding both professional judgment and patient trust in human clinical skills.

The surveillance dimensions of DTs raise particular concerns about social control and childhood development. Drummond and Coulet (2022, p. 8) envision troubling scenarios where continuous monitoring creates “a surveillance society” where “children would grow up in an optimized state of physical health but would probably be more anxious, dependent, and conventional in adulthood.” This analysis highlights how DTs could fundamentally alter human development and social relationships, creating populations accustomed to constant health monitoring and algorithmic guidance. An aforementioned point by Winter and Chico (2023, p. 12) compounds these concerns by noting commercial exploitation risks where “patient data could be used primarily by companies as a commercial tool,” potentially undermining trust through perceived exploitation of health information for profit rather than care improvement.

##### Knowledge and advancement cluster

DTs promise to accelerate public health knowledge generation and healthcare advancement, yet this potential comes with risks of cognitive offloading and expertise erosion. de Kerckhove (2021, p. 7) identifies a broader pattern: “the more people trust their faculties to machines, the less they tend to exercise them themselves. the externalization of most of our cognitive abilities, beginning with the delegation of our memory function to recording devices”.^12^ In healthcare contexts, this could manifest as diminished clinical reasoning skills, reduced diagnostic capabilities, and weakened capacity for complex clinical judgment that relies on accumulated experiential knowledge.

The literature suggests that DTs could democratise medical knowledge by making sophisticated analytics accessible to non-specialists, potentially empowering patients and improving health literacy. However, this democratisation raises questions about knowledge quality and interpretation. Complex population health insights generated through DT aggregation could inform policy decisions and public health interventions, yet the opacity of algorithmic processes may obscure important limitations or biases in these insights. The promise of advancing medical knowledge through DTs must be balanced against risks of oversimplification, algorithmic bias, and the potential loss of nuanced clinical expertise that emerges from direct patient care experience.

Environmental sustainability emerged as a singular minor social theme, discussed in the Supplementary Material (Sect.  3).

#### Gaps

While 95% of rare diseases lack approved treatments (Lancet Global Health 2024), DT applications for these conditions remain virtually absent from social impact frameworks, creating a cruel irony where approximately 300 million people globally affected by rare diseases face exclusion from technologies designed for personalization (World Health Organisation 2025). The literature’s focus on common conditions and well-represented populations ignores how DTs risk becoming tools of ‘digital colonialism’,—a concept describing how digital technologies can reproduce colonial power structures by imposing dominant cultural frameworks without adequate consideration of local contexts (Kwet 2019).

It is important, therefore, to note that while this represents a gap in the healthcare DTs literature specifically, it is far from being a gap in the biomedical ethics literature. The broader literature on global health ethics and justice, including frameworks such as the UN sustainable development goals, encompasses data-driven technologies like DTs within wider discussions of health equity, technology transfer, and capacity building in global health systems (Venkatapuram and Marmot 2013). This suggests that the DT literature would benefit from engaging with established global health ethics frameworks rather than developing isolated approaches.

### Barriers to the development and implementation of DTs

Barriers determine whether healthcare DTs become clinical realities. Our analysis identified six major themes in discussions of practical implementation barriers: data challenges, human factors, integration and standardisation, model fidelity and validation, technical infrastructure and scalability, and cybersecurity. These barriers are deeply interconnected, 13 meaning implementation challenges cannot be addressed in isolation. We discuss this further in the next section.

#### Major themes

##### Data challenges

Data represents both the driving force and foundational barrier to successful healthcare DTs. Winter and Chico (2023, p. 11) note that even with data access, “standardised approaches to data fusion are currently lacking”, and “patient data needs integration into local human and organisational ecosystems from decentralised sources”. This technical challenge directly impacts patient consent and autonomy—when data from multiple sources are fused, patients lose visibility into how their information is being combined and used. This extends beyond technical interoperability to organisational workflows and governance frameworks evolved independently of DT requirements. Healthcare data cannot be seamlessly plugged into DT systems given decades of fragmented development, raising questions about accountability when errors arise from data integration failures.

Data quality problems create cascading challenges. Effective DT operation requires cleaning data while maintaining accuracy and quality. Yet healthcare data carries inherent messiness—missing values, inconsistent terminology, temporal misalignment. Drummond and Coulet (2022, p. 4) offer an example of this challenge from paediatric populations where “data available is lower due to young age (less historical data), logistical and legal difficulties,” compromising statistical power. DTs require comprehensive, continuous data streams, yet healthcare generates fragmented, episodic, heterogeneous data resisting standardisation.

##### Human factors

Human factors encompass trust, usability, cognitive compatibility, and mismatches between human and algorithmic logic 14 —issues central to NASSS ‘adopter’ capabilities (dimension 4). Dahir et al. (2023, p. 83) note: “There is often a great deal of human intuition involved. machine logic doesn’t always jibe with the human brain.” This disconnect also manifests differently across stakeholders: clinicians struggle with recommendations contradicting experience, patients resist invasive monitoring, administrators grapple with workflow disruptions reducing short-term efficiency, all affecting ‘organisational readiness’ (dimension 5) for DT implementation.

The complexity multiplies when considering the sociotechnical nature of healthcare delivery, where DTs must integrate with existing professional practices, institutional cultures, and patient preferences. Drummond and Coulet (2022, p. 4) offer an example where “connected inhalers that automatically record children’s use of asthma medication were reported lost or damaged by up to 50% of families,” demonstrating how real-world usage patterns can diverge dramatically from laboratory assumptions. The literature often treats human factors as implementation details to be managed or risks to be mitigated, rather than fundamental design constraints that shape what DT functionalities are feasible. This perspective fails to recognise that successful DT adoption requires much deeper alignment with human cognitive processes, professional workflows, and social relationships than the literature acknowledges.

##### Integration and standardisation

The integration and standardisation barrier reveals a gap between DT aspirations and healthcare realities, encompassing technical interoperability and organisational coordination across fragmented ecosystems (see Table 1). Chase et al. (2023, p. 460) articulates the fundamental problem: “What is missing is a collection of accurate, validated virtual patient or DT models for use at the bedside. there are no accepted standards for modeling approach, model identifiability, or accepted levels of model validation.” (For detailed discussion of validation challenges, see model fidelity and validation below.) This standards vacuum creates cascading barriers with profound ethical implications. Without common modelling approaches, DTs remain incompatible across organisations, potentially creating disparities in care quality; without validation standards, clinicians cannot assess reliability, undermining informed consent and professional liability frameworks; and without identifiability standards, models cannot be verified, eroding the evidence base required for regulatory approval and clinical trust.

While formal standards exist (IEEE, ISO, HL7), the challenge extends beyond data exchange to encompass the complex web of devices, systems, and processes constituting modern healthcare. Even established standards, such as DICOM for imaging or SNOMED CT for terminology, primarily address static data exchange rather than DTs’ dynamic, real-time requirements. Sharma et al. (2023, p. 1317) acknowledges that “Managing the vast number of linked devices, fixing security holes, and ensuring interoperability are the three main IoT challenges.”

To be successful, DTs must bridge these heterogeneous systems while maintaining security, reliability, and regulatory compliance—a challenge that grows exponentially with each integrated system. Much of the literature overlooks these integration challenges, yet decades of healthcare interoperability efforts demonstrate that technical solutions alone cannot overcome organisational, economic, and political barriers to standardisation.

##### Model fidelity and validation

Model fidelity and validation represents both a technical and epistemic barrier, although the literature primarily treats it as technical (Chase et al. 2023). Popa et al. (2021, p. 20) provide complementary perspective: “if we notice that it took a long time to arrive at a fair understanding of airplane engines (which are, in essence, fairly simple rotary machines), we should expect the DT to be exponentially more difficult because any human organ is indefinitely more complex than the most complex aeroplane engine.” DT validation exceeds anything previously attempted, requiring models capturing not just organ function but dynamic interactions between biological systems, psychological states, and environmental factors.

Determining ‘sufficient fidelity’ (i.e. what level of detail is necessary for the specified task) is subjective, context-dependent, and often influenced by a wide range of sociotechnical forces that can remain invisible, such as organisational and cultural processes. This subjectivity raises ethical questions about who decides acceptable accuracy levels for life-critical decisions and how patients can provide meaningful consent when model limitations remain uncertain. And, unlike engineering systems with clear performance specifications, biological systems exhibit emergent properties that may resist computational capture at available fidelity levels, creating potential liability issues when simplified models fail to predict rare but serious complications.

The fidelity and validation challenge is compounded by the personalisation imperative that distinguishes DTs for personalised medicine from population-level models (see Sect.  2). As (Chase et al. 2023, p. 460) notes, “the lack of accurate, implementable virtual patient or DT models is the primary technical and scientific hurdle to linking measurements and care delivery devices to automate care in an accurate, personalized fashion”. Traditional validation approaches often rely on population studies and averaged effects, yet a key promise of healthcare DTs is their demonstrable validity and efficacy for individual patients whose unique characteristics may fall outside training data distributions. This creates a fundamental tension between statistical validation approaches that require large datasets and personalisation requirements that demand individual-specific accuracy.

##### Technical infrastructure and scalability

Technical infrastructure and scalability barriers concretely manifest the promise-reality gap. Dahir et al. (2023, p. 88) emphasise how “continuous data streams are needed and the more of the relevant data is captured, the easier, faster, and more reliable it becomes for us to build a DT model”. Yet this requirement clashes with healthcare realities—intermittent connectivity, limited bandwidth, ageing hardware, and constrained ICT budgets that cannot support real-time DT operations.

The scalability challenge extends beyond infrastructure to computational and economic sustainability. Vallée (2024, p. 8) offer an example of the computational barrier, noting that “conducting sensitivity analysis on large-scale simulations becomes computationally challenging due to the numerous parameters involved”. This computational burden necessitates developing surrogate models or emulators that can operate on limited hardware while maintaining clinical validity. However, these pipelines require sophisticated technical expertise, creating additional barriers for healthcare organisations lacking dedicated computational teams.

##### Cybersecurity and data security

Cybersecurity and data security barriers represent mature challenges for all safety–critical national infrastructure (cf. ‘Privacy Protection’ row in Table 1). And, unsurprisingly, healthcare DTs amplify these concerns because of the highly sensitive data that are aggregated and potentially transmitted across sites or areas of the public and private sector. Popa et al. (2021, p. 14) warns that “the advent of a DT can amplify (rather than solve) the hazards of data being lost, leaked or stolen through security breaches or negligence. In a scenario of increased data gathering through digitalization, the depth, and sensitivity of information that ‘ends up on the street’ is much more significant than in the case of the age-old digital patient file”. As DTs create comprehensive, dynamic profiles that combine physiological monitoring, behavioural tracking, genetic information, and predictive analytics into integrated datasets (i.e. digital threads), they represent unprecedented security risks if compromised.

The challenge extends beyond data protection to encompass system integrity and availability requirements that DTs demand. Unlike traditional medical records that can tolerate temporary access disruptions, DTs require continuous connectivity and real-time data processing to maintain clinical utility. This creates attack surfaces and dependency relationships that malicious actors can exploit to disrupt care delivery or manipulate clinical decisions through compromised models. Sharma et al. (2023, p. 1318) identify the scope of required improvements, noting that “research should focus on efficient data management, robust security measures, flexible technical solutions, and advancements in AI”. Yet healthcare cybersecurity already struggles with basic protection of static records. Expanding to protect dynamic, interconnected DT systems requires security capabilities and investment levels that exceed current healthcare capacity.

Three minor barrier themes—regulatory and legal gaps, collaboration and engagement, and economic and commercial barriers—are detailed in the Supplementary Material (Sect.  3). Rather than addressing gaps in this thematic category here, we now turn to a more focused proposal that connects these barriers to the NASSS framework and offer targeted recommendations.

## Practical recommendations

While much of the literature catalogues risks and opportunities, few studies identify the key barriers to mitigating those risks or realising potential opportunities. To address this gap, we map our identified barriers onto the 7 domains of the nonadoption, abandonment, scale-up, spread, sustainability (NASSS) framework (Greenhalgh et al. 2017), grounding our recommendations in established implementation science.

**Fig. 5:**
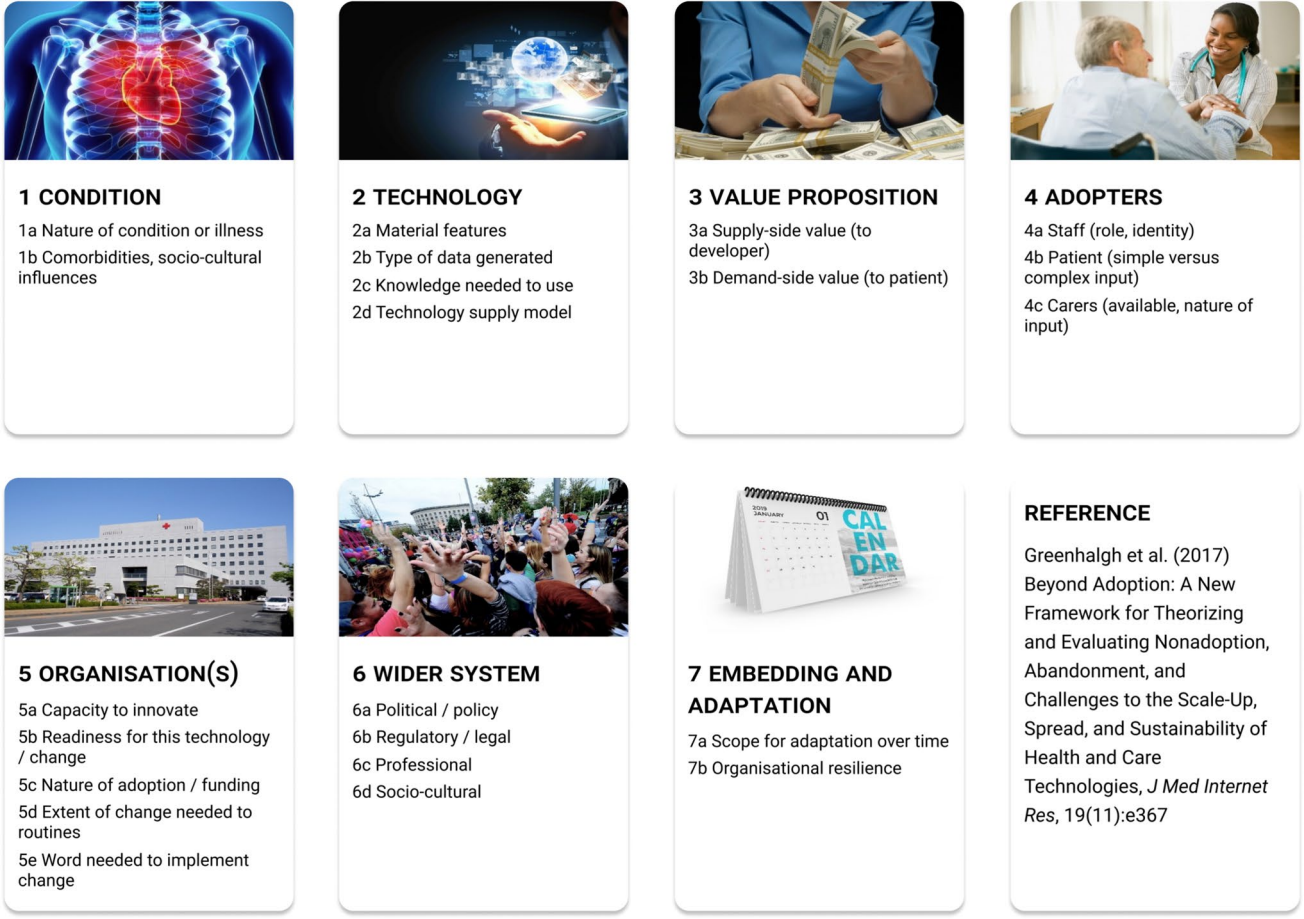
The NASSS framework’s seven domains for evaluating healthcare technology implementation. Each domain captures different dimensions of complexity that influence whether innovations are successfully adopted, scaled, and sustained in healthcare settings

The NASSS framework helps healthcare practitioners understand why health technologies succeed or fail. As shown in Fig. 5, the framework comprises seven domains. Domains 1–3 address foundations: The condition (illness complexity), the technology (technical features and maturity), and the value proposition (affordability and value). Domains 4–5 focus on adoption: The adopter system (staff and patient uptake) and the organisation (institutional capacity and readiness). Domains 6–7 address context and evolution: The wider system (regulatory and sociocultural context) and embedding and adaptation over time (technology evolution within dynamic health systems).

Mapping our identified barriers (Sect. 4.5.1) to NASSS domains serves three purposes. First, it recontextualises our 6 barriers within a proven framework for understanding healthcare technology failures. Second, it extends NASSS by connecting practical barriers to their ELSI foundations. Our analysis reveals how normative dimensions permeate all domains, from epistemic credibility in technology validation (Domain 2) to justice and accountability in organisational readiness (Domain 5). Third, by linking barriers to NASSS domains while maintaining ELSI visibility, we enhance the understanding of the promise-reality gaps (Table 1) and enable stakeholder-specific recommendations for addressing them. The stakeholder groupings we focus on are:

1. **Policy and regulation**: data governance professionals, auditors, policy-makers, and regulators
2. **Healthcare**: healthcare professionals, patient engagement
3. **Research and development**: academics, software engineers, public and private sector researchers

The recommendations we provide have critical inter-dependencies and foundational prerequisites that align to three of the barriers mentioned in the previous section: (1) technical infrastructure for DT operations (technical infrastructure and scalability); (2) data governance and quality standards for model building (data challenges); (3) integration frameworks for cross-institutional collaboration (integration and standardisation). As such, we present the barriers in this revised order rather than the order from the preceding section.

### Technical infrastructure and scalability

#### Primary mapping: Domain 5 (the organisation)

Greenhalgh et al. (2017) remind us that organisational factors often determine whether an innovation is sustained, abandoned, or transformed in practice. DTs in healthcare infrastructures bring these tensions to the fore.

In terms of capacity to innovate (5A), most healthcare organisations operate closer to the complicated or complex end of the spectrum. Severe resource pressures, frozen posts, and overstretched IT departments leave little slack to experiment with continuous, data-intensive infrastructures. Leadership is often reactive rather than visionary, with risk-taking discouraged by financial constraints and fear of reputational damage if systems fail. This organisational fragility is likely to shape whether DT projects remain pilots or move to broader implementation.

Readiness for change (5B) is similarly limited. While proponents may speak of the ‘inevitability’ of precision or AI-enabled medicine, healthcare organisations continue to struggle with systemic change. Against this backdrop, DTs appear poorly aligned with institutional trajectories. Opposition often arises from both clinicians and administrators who are likely to perceive DTs as misfits within current workflows—what Greenhalgh et al. (2017, p. 12) describe as a poor “innovation-system fit”.

Adoption and funding decisions (5C) reflect this organisational inertia. The literature suggests that DTs are likely to require multiple organisations to coordinate, yet these entities frequently lack formal links and operate under conflicting political agendas. Funding is further complicated because the costs (e.g., hardware, specialist staff, cybersecurity upgrades) accrue locally, while benefits (e.g., predictive insights, population-level efficiencies) may be diffuse or long-term. This tends to lead to contested decision-making in which budget-holders are reluctant to commit.

Scaling also entails major changes in team interactions and routines (5D). DTs do not simply ‘slot into’ existing care pathways, they demand new practices of interpretation, validation, troubleshooting, and repair that often conflict with established routines. For instance, clinicians accustomed to episodic decision-making must adapt to continuous data feeds, while IT staff assume quasi-clinical responsibilities for ensuring model integrity. Such changes risk destabilising existing professional boundaries and hierarchies.

Finally, implementation work (5E) is both intensive and contested. Building a shared vision across diverse stakeholders requires sustained engagement and negotiation. Considerable labour is involved in data acquisition, collection, cleaning, training, testing, model calibration, and infrastructural maintenance, much of which is invisible and unrecognised in formal resource allocations. Without explicit recognition of this invisible work, organisations risk underestimating what is required for sustained operation.

#### Gaps and recommendations

This mapping helps to highlight how the promise of DTs is mediated not only by technical feasibility but also by the material and organisational infrastructures in which they are embedded. Infrastructure investments would help bridge the ‘economic value’ gaps (see Table 1), with cloud migration offering quick efficiency gains while comprehensive HPC investments represent long-term structural improvements necessary for population-scale deployment. Shared infrastructure models also help to address socioeconomic barriers (Sect. 4.3) by enabling resource pooling for smaller institutions. Our specific recommendations are:

##### Policy and regulation

- Develop risk-based certification frameworks that set minimum infrastructural requirements for safe DT deployment (e.g., readiness and bandwidth, computational capacity).
- Establish clear accountability mechanisms for infrastructure-related failures and performance degradation.

##### Healthcare

- Allocate protected time (e.g. 4 h per quarter) for the hidden work of implementation.
- Deliver training through existing quality improvement frameworks, prioritising skills for managing continuous data streams.

##### Research and development

- Design multi-site pilot studies following CONSORT-AI protocols with mandatory sharing of implementation blueprints (Liu et al. 2020).
- Advance computationally efficient models with open benchmarking datasets and standardised performance metrics.
- Document implementation patterns using established frameworks.

### Data challenges

#### Primary mapping: Domain 2 (the technology)

DTs are complex systems (2A) requiring continuous, high-quality data from heterogeneous sources, yet healthcare infrastructures remain fragmented with inconsistent standards, making dependability a persistent issue (Winter and Chico 2023). For knowledge brought into play (2B), DTs again fall into the complex category. Their knowledge is not directly or transparently produced, but constructed through processes of data fusion, cleaning, and modelling. The resulting outputs are often uncertain, contested, or only partially linked to clinical outcomes (Popa et al. 2021). For example, in paediatrics, limited historical data and legal constraints create sparse, fragmented inputs, requiring additional modelling assumptions that reduce transparency (Drummond and Coulet 2022). Thus, the credibility of DTs rests on negotiated judgments about what constitutes ‘sufficient’ data, reflecting both technical and epistemic uncertainties.

#### Gaps and recommendations

Although the ‘data challenges’ barrier maps most directly onto Domain 2 (the technology), there is also overlap with Domain 5 (the organisation), as institutional governance structures, entrenched information infrastructures, and clinical workflows critically shape how data can be mobilised in practice.

The following recommendations help address the ‘Seamless Integration’ gap (see Table 1 above) by establishing data standards and interoperability frameworks, while policy measures and architectural changes target different implementation timelines. The privacy-preserving frameworks specifically address surveillance concerns (Sect. 4.1) while GDPR-compliant governance ensures autonomy protections through explicit consent mechanisms.

##### Policy and regulation

- Establish risk-stratified standards for data quality and interoperability following medical device classifications (low/moderate/high risk), recognising that DTs will predominantly fall into moderate-to-high risk categories (European Medicines Agency 2024; Medicines and Healthcare products Regulatory Agency 2025).
- Mandate ongoing post-market audits to monitor data performance under real-world conditions.
- Support privacy-preserving frameworks (federated learning, differential privacy, homomorphic encryption) that balance innovation with GDPR-compliant governance (Voigt and Bussche 2024).

##### Healthcare professionals

- Develop context-specific thresholds for data quality that clinicians can apply in practice, acknowledging time constraints by prioritising training on critical data quality indicators most relevant to clinical decision-making.
- Co-produce validation metrics with patients through existing quality improvement frameworks, balancing statistical sufficiency against individual-level clinical utility.
- Collaborate with IT specialists and data stewards to embed DT data flows into everyday workflows, with protected time allocation of 2–4 h per month for familiarisation and workflow integration.

##### Research and development

- Design modular architectures for data fusion that accommodate heterogeneous sources following FAIR data principles.
- Develop standardised documentation protocols for data lineage using TRIPOD + AI reporting guidelines (Popa et al. 2021; Cohen and Bossuyt 2024).
- Invest in scalable methods such as surrogate modelling and data-cleaning pipelines.

### Integration and standardisation

#### Primary mapping: Domain 6 (the wider system)

According to the NASSS framework, technologies succeed or fail within broader policy, regulatory, and professional environments. Integration challenges highlight the problem: without common standards for how models are built, validated, and shown to be identifiable—meaning their parameters can be uniquely tied to patient data—DTs will remain fragmented across institutions (Chase et al. 2023). Sharma et al. (2023) note that DTs must bridge heterogenous infrastructures (e.g. IoT devices, imaging systems, and health information exchanges) creating dependencies that extend beyond any single organisation’s control. This aligns with NASSS questions 6A (policy/regulation) and 6C (professional bodies), where the absence of consensus undermines both regulatory pathways and professional confidence. Winter and Chico (2023) demonstrate that without coordinated standards, DT projects are likely to stall at pilot stage, unable to scale sustainably.

#### Gaps and recommendations

The ‘integration and standardisation’ barrier also overlaps with Domain 2 (the technology), since DTs are technically complex systems requiring real-time, multi-scale interoperability that existing healthcare standards were not designed to support. While often presented as technical interoperability issues, integration and standardisation challenges are fundamentally systemic. Without agreed-upon standards for validation, identifiability, and modelling approaches, DTs risk being siloed across organisations, stalling at the pilot stage rather than achieving sustainable scale. Developing these standards would help to address the moderate gap in ‘Interoperability’ and help reduce ‘regulatory complexity’ (see Table 1), with harmonised certification processes and predetermined change control plans (PCCPs) providing quick wins while cross-jurisdictional standards alignment represents long-term structural change. Our specific recommendations for this mapped barrier are as follows:

##### Policy and regulation

- Establish cross-jurisdictional working groups to harmonise standards for interoperability, validation, and identifiability through bodies like the International Medical Device Regulators Forum (IMDRF).
- Introduce adaptive regulatory pathways leveraging PCCPs that recognise the dynamic nature of DT systems, building on HL7 FHIR and ISO frameworks but extending them for continuous data integration and model evolution.
- Develop funding mechanisms and policy incentives to encourage cross-sector adoption of common standards, reducing fragmentation across healthcare providers.

##### Healthcare professionals

- Engage clinicians and professional communities in the co-design of interoperability standards through existing quality improvement cycles, ensuring alignment with clinical workflows and maintaining professional accountability.
- Given time constraints, integrate interoperability training into mandatory information governance sessions, focusing on practical skills for managing data exchange between systems.
- Support local champions through existing clinical leadership programmes.

##### Research and development

- Prioritise dynamic standard frameworks moving beyond static data exchange towards real-time continuous interoperability.
- Encourage open data and model repositories following FAIR principles for cross-institutional validation.
- Develop reference implementations with complete documentation under permissive licenses.

### Model fidelity and validation

#### Primary mapping: Domain 2 (the technology)

Domain 2 addresses feasibility, maturity, and uncertainty of healthcare technologies—issues that crystallise in fidelity-validation debates (Committee et al. 2024). With respect to their key features (2A), DTs are complex (e.g. relying on multi-scale simulations that integrate physiological, behavioural, and contextual factors). Yet their dependability remains fragile, since validation frameworks are still immature (Popa et al. 2021). The kind of knowledge DTs bring into play (2B) is similarly shaped: outputs are not direct readings of a patient’s condition but mediated through modelling choices, criteria, terms of comparison, and proxies. Fidelity may render these outputs clinically meaningful—or expose them as uncertain and misleading. Validation is what stabilises these knowledge claims, but as Chase et al. (2023, p. 460) note, it remains the “primary technical and scientific hurdle”.

The knowledge and support required to use the technology (2C) likewise reflects this instability. Without open and shared validation benchmarks, clinicians and organisations must interpret and negotiate outputs in practice, requiring both technical expertise and new epistemic routines. Finally, the supply model (2D) is also shaped by these barriers: DTs are delivered through bespoke, project-based arrangements with no common certification or benchmarking (Fuller et al. 2020), situating them firmly at the ‘complex’ end of the NASSS spectrum.

#### Gaps and recommendations

This theme has relevant crossovers into Domain 3 (the value proposition). The following recommendations target the HIGH gap in ‘Personalised Medicine’ (Table 1) by establishing validation frameworks and developing comprehensive accuracy metrics, while addressing transparency deficits that undermine clinical trust. Transparency requirements and explainability standards also directly address accountability concerns (see Sect. 4.1), while bias mitigation in validation specifically targets algorithmic discrimination risks (see Sect. 4.1).

##### Policy and regulation

- Establish risk-stratified validation frameworks for healthcare DTs aligned with medical device classifications, recognising that sufficient fidelity will vary by clinical context and use case (Bruynseels et al. 2018).
- Implement transparency principles from MHRA, FDA, and Health Canada for ML-enabled medical devices (Medicines and Healthcare products Regulatory Agency 2024), adapted for DTs to include modelling assumptions, data provenance, validation methods, and infrastructural limitations.
- Support adoption of Predetermined Change Control Plans (PCCPs) enabling manufacturers to pre-specify and validate planned modifications (Medicines and Healthcare products Regulatory Agency 2023).

##### Healthcare professionals

- Given clinical workload constraints, deliver focused 2 h quarterly (or bi-annually) training sessions to equip clinicians with skills for interpreting model limitations and uncertainty metrics.
- Implement patient communication through established consultation frameworks, with templates adapted from NHS shared decision-making resources to explain model validation status and uncertainty (Elwyn et al. 2017; England 2025).

##### Research and development

- Conduct comparative validation studies following TRIPOD + AI reporting standards with pre-registration and mandatory data sharing (Chase et al. 2023; Cohen and Bossuyt 2024).
- Develop context-specific benchmarks with open-source validation datasets and standardised evaluation protocols.
- Explore hybrid approaches (surrogate models, emulators) with reproducible research practices.

### Human factors

#### Primary mapping: Domain 4 (the adopter system)

Healthcare technology adoption is shaped not only by a technology’s properties but also by how staff, patients, and carers make sense of it in practice. The human–machine mismatches described in Dahir et al. (2023) and Drummond and Coulet (2022) highlight how DTs may clash with experiential knowledge, expectations of care, or social routines. Clinicians may reinterpret or resist algorithmic outputs that appear counterintuitive; patients may reject continuous monitoring when it feels invasive; and carers may adapt or circumvent devices in unanticipated ways.

These dynamics exemplify what Greenhalgh et al. (2017, p. 9) call ‘coherence work’—the labour required to render technologies meaningful and usable in everyday contexts. They also reflect broader ethnographic insights into how technologies must be “tamed” or “domesticated” in practice, often requiring substantial hidden work (Pols and Willems, 2011, as cited in Greenhalgh et al. 2017, p. 13).

#### Gaps and recommendation

The following recommendations target the MODERATE gap in ‘Enhanced Efficiency’ (Table 1) through training initiatives (quick wins) and workflow integration (long-term), while also addressing trust and autonomy concerns that contribute to adoption challenges. They also address autonomy and agency concerns (Sect. 4.1) through patient co-production and the equity challenges (Sect. 4.1) by ensuring diverse stakeholder involvement in system design.

##### Policy and Regulation

- Develop clear guidelines on algorithmic accountability clarifying roles when clinicians override DT outputs (Lawton et al. 2024).
- Establish participatory mechanisms for patients and carers in policy-making to ensure public trust (Popa et al. 2021).
- Require manufacturers to include usability testing and human factors evaluations (European Medicines Agency 2024; Medicines and Healthcare products Regulatory Agency 2025).

##### Healthcare professionals

- Given time constraints, prioritise training on interpreting uncertainty metrics and override protocols, combining technical literacy with interpretive flexibility to help clinicians integrate DT recommendations alongside experiential knowledge.
- Embed patient communication frameworks that acknowledge concerns around monitoring, intrusiveness, and autonomy, utilising established shared decision-making models and NHS Personalised Care resources adapted for DT contexts (Bruynseels et al. 2018; Elwyn et al. 2017; England 2025).
- Establish comprehensive multidisciplinary teams through existing clinical governance structures, with protected time of 4 h per month for team members to: (a) collaboratively adapt DT workflows into practice, (b) negotiate context-specific thresholds for model fidelity and clinical use, and (c) align infrastructure upgrades with workflow changes.

##### Research and development

- Prioritise real-world usability studies following CONSORT-AI guidelines with mandatory data sharing (Drummond and Coulet 2022; Liu et al. 2020).
- Investigate trust calibration mechanisms through pre-registered studies with open protocols (Burton et al. 2020).
- Develop participatory research models with patient/public involvement reporting.

### Cybersecurity and data security

#### Primary mapping: Domain 6 (the wider system)

Cybersecurity and data protection sit squarely at the intersection of political, regulatory, professional, and sociocultural contexts—all of which represent Domain 6 of NASSS. Legal obligations are set by supranational and national regimes, such as the GDPR in Europe (Voigt and Bussche 2024), the MHRA’s guidance on software and AI as a medical device (Medicines and Healthcare products Regulatory Agency 2025), and the FDA’s assurance frameworks (US Food and Drug Administration 2022). But beyond regulation, cybersecurity is bound up with public confidence and professional liability. Popa et al. (2021) note that DTs can amplify hazards of data loss or breaches, while Lawton et al. (2024) highlight clinicians’ fears of becoming “liability sinks” for AI-related failures.

These concerns reflect not only technical risks but also broader sociopolitical anxieties about surveillance, accountability, and trust in DT infrastructures (Winter and Chico 2023). Following Greenhalgh et al. (2017), the issue can be considered complex—policies remain contested, liabilities are unclear, and societal expectations about what constitutes ‘secure enough systems’ vary across jurisdictions and publics.

#### Gaps and recommendations

Cybersecurity and data security are well-known but still evolving challenges, and they become especially important in healthcare. Because DTs bring together highly sensitive, constantly updated data from different organisations, they create more opportunities for attacks on health systems. This heightens concerns around trust, liability, and oversight.

The ‘Cybersecurity and Data Security’ theme also links to Domain 2 (the technology), as technical design and supply models determine how secure these systems can be. These security measures address critical vulnerabilities that could undermine all promise areas (Table 1), with immediate security audits providing quick wins while comprehensive zero-trust architectures and quantum-resistant cryptography represent long-term structural protections against evolving threats. Privacy-preserving techniques and incident response protocols also address surveillance society concerns (Sect. 4.3), while ensuring patient autonomy through granular consent controls.

##### Policy and regulation

- Update national and international cybersecurity standards specific to real-time, dynamic systems like DTs.
- Address jurisdictional risks linked to extraterritorial laws such as the US CLOUD Act by requiring contractual safeguards and sovereign solutions.
- Clarify liability regimes so responsibility for breaches is appropriately distributed (Lawton et al. 2024).
- Mandate independent security audits and post-market monitoring.
- Support public engagement on data protection in DTs.

##### Healthcare professionals

- Integrate security training into mandatory information governance sessions, focusing on practical identification of vulnerabilities in DT workflows.
- Build collaborative governance through existing clinical governance committees.
- Engage patients using adapted NHS communication templates to transparently discuss cybersecurity risks and mitigation strategies (England 2025).

##### Research and development

- Develop security-by-default architectures with threat models shared via OWASP repositories.
- Design simulation-based ‘red teaming’ exercises with anonymised attack scenarios.
- Investigate sociotechnical dynamics through mixed-methods research.

These recommendations provide actionable pathways for realising the potential of healthcare DTs while managing their risks. In the following conclusion, we synthesise the key insights from our analysis and reflect on the broader implications for the future of this transformative technology.

## Conclusion

Our analysis revealed a persistent gap between DT aspirations and capabilities, with fundamental ethical, legal, and social challenges underpinning implementation barriers. Through systematic thematic analysis of 29 papers, we identified eleven major themes spanning privacy concerns, bias and discrimination, data governance challenges, and cybersecurity vulnerabilities. These findings demonstrate that healthcare DTs face not merely technical hurdles but profound sociotechnical challenges requiring coordinated responses across multiple stakeholder groups.

The integration of our findings with the NASSS framework reveals critical interdependencies. Privacy concerns and algorithmic bias directly impact the ‘value proposition’ dimension by undermining trust. Data governance challenges affect ‘organisational readiness’ by requiring new institutional capabilities. Cybersecurity vulnerabilities threaten ‘systemic’ stability. These connections demonstrate that ELSI considerations are not peripheral compliance issues but fundamental determinants of technology adoption and sustainability.

For policy-makers, our analysis highlights the urgent need for adaptive regulatory frameworks that can evolve alongside continuously learning systems. Current regulatory paradigms, designed for static medical devices, cannot adequately govern DTs that continuously adapt through machine learning. For healthcare professionals, the findings emphasise the importance of maintaining clinical judgement while integrating algorithmic insights, requiring new forms of digital literacy and collaborative decision-making frameworks.

Our interdisciplinary approach bridges technical and normative considerations, revealing how ethical, legal and social issues permeate many facets of practical implementation barriers. And, our recommendations address these barriers through proportionate regulation, context-specific validation, and collaborative patient engagement. Going forward, success will require building systems that patients trust, clinicians can effectively use, and societies can equitably govern.

We must resist technological solutionism and hype, instead building trust through transparency, ensuring equity through socially and politically inclusive design, and maintaining patient dignity through governance. In pursuing precision medicine’s ideal, we must not lose sight of healthcare’s imperative to do good.

## Supplementary Information

Below is the link to the electronic supplementary material.

Supplementary file1 (DOCX 6615 kb)

## Data Availability

No datasets were generated or analysed during the current study.

## Footnotes

1 We designed this taxonomy to support our thematic analysis, not as a taxonomy for all healthcare DTs more broadly.

2 In the UK regulatory framework, in silico trials serve as enhanced preclinical studies or exploratory Phase 0 trials. The MHRA increasingly supports model-informed drug development (MIDD) approaches, though human participation remains essential for Phase I-III trials to validate safety and efficacy (Kerwash 2025). Virtual populations help optimise trial design and reduce patient numbers needed in early phases, aligning with the UK’s commitment to the 3Rs (replacement, reduction, and refinement) principle.

3 Federated learning alternatives exist where data remains distributed and only model parameters are shared.

4 Discrete-event simulation models systems as time-sequenced events; agent-based simulation represents entities as autonomous agents with behaviours and decision rules.

5 A citation network analysis tool for discovering relevant papers (https://www.researchrabbit.ai/).

6 These ‘Gap’ assessments shown in this table are not derived from the literature; they reflect our own judgements. LARGE = fundamental challenges requiring breakthroughs; MEDIUM = substantial challenges with visible progress pathways.

7 See (Barocas et al. 2023).

8 While strongly overlapping with the related ethical theme, ‘privacy and data governance’ (see Sect. 4.2.1), it is important to separate ethical and legal discussions concerning data privacy and protection. The former typically focuses on broad, normative issues, while the latter presumes these foundations (often implicitly) in favour of practical considerations.

9 The Irish data protection Commission’s 2025 €530 million fine against TikTok for transferring EEA user data to China demonstrates these tensions, with the DPC finding that Chinese authorities’ potential access to data under national security laws materially diverges from EU standards (Data Protection Commission 2025). See also Bradford (2023) for analysis of fundamental incompatibilities between Chinese and European approaches to data governance.

10 It could also create what (Lawton et al. 2024) term a “liability sink," where clinicians risk absorbing legal accountability for AI-driven decisions without sufficient understanding or practical control over algorithmic recommendations.

11 Here ‘social license’ refers to the ongoing acceptance of an organisation’s standard business practices and operating procedures by its employees, stakeholders, and the general public, achieved through transparent engagement and demonstrated commitment to shared values.

12 For further discussion and analysis of cognitive offloading on to DTs, see (Burr et al. 2025).

13 Co-occurrence analysis revealed 99.1% connectivity between barriers and issues.

14 See (Burton et al. 2020) for a discussion of algorithmic aversion that explores these topics.
