# Supplementary material for: Realising the digital twin: a thematic review and analysis of the ethical, legal, and social issues for digital twins in healthcare
Source: AI Soc. 2026 Feb 7;41(5):5243–67. doi: 10.1007/s00146-025-02833-6 (PMC13241403; doi:10.1007/s00146-025-02833-6)
Supplement: Supplementary file 1 — Supplementary file1 (DOCX 6615 kb) [file 146_2025_2833_MOESM1_ESM.docx]

Supplementary Material—Realising the Digital Twin: A Thematic Review and Analysis of the Ethical, Legal, and Social Issues for Digital Twins in Healthcare

Anonymised for peer review

2025-11-18

# Overview

This supplementary material contains:

- **Section** [**2**](#sec:methodology-detail): Comprehensive methodology details including the seven-stage review process, search results tables, and codebook development
- **Section** [**3**](#sec:minor-themes): Detailed analysis of minor themes identified in our thematic review but not included in the main manuscript for space considerations
- **Section** [**4**](#sec:supp-limitations): Methodological limitations and considerations

# Detailed Methodology

This section provides a comprehensive account of the seven-stage methodology that is summarised in the main paper:

1. **Literature Search:** a systematic literature search across multiple databases (i.e. IEEE, PubMed, ScienceDirect),
2. **Filtering:** filtering to determine relevant sources,
3. **Augmentation:** augmentation of initial search results via network analysis (i.e. ResearchRabbit),
4. **Independent Coding:** independent review and coding of the papers by three reviewers using an emergent codebook approach,
5. **Consensus Formation:** collaborative consensus formation to refine and consolidate 182 initial codes (version 1) to 34 consolidated themes (version 2),
6. **Mixed-Methods Analysis:** detailed analysis was conducted to identify robust patterns in the codes and help split them into major and minor themes.
7. **Theme Definition and Analysis Write-Up:** definition of the updated major and minor themes, using original annotations to help provide additional context.

These stages are shown in Figure [1](#fig:methodology-stages).


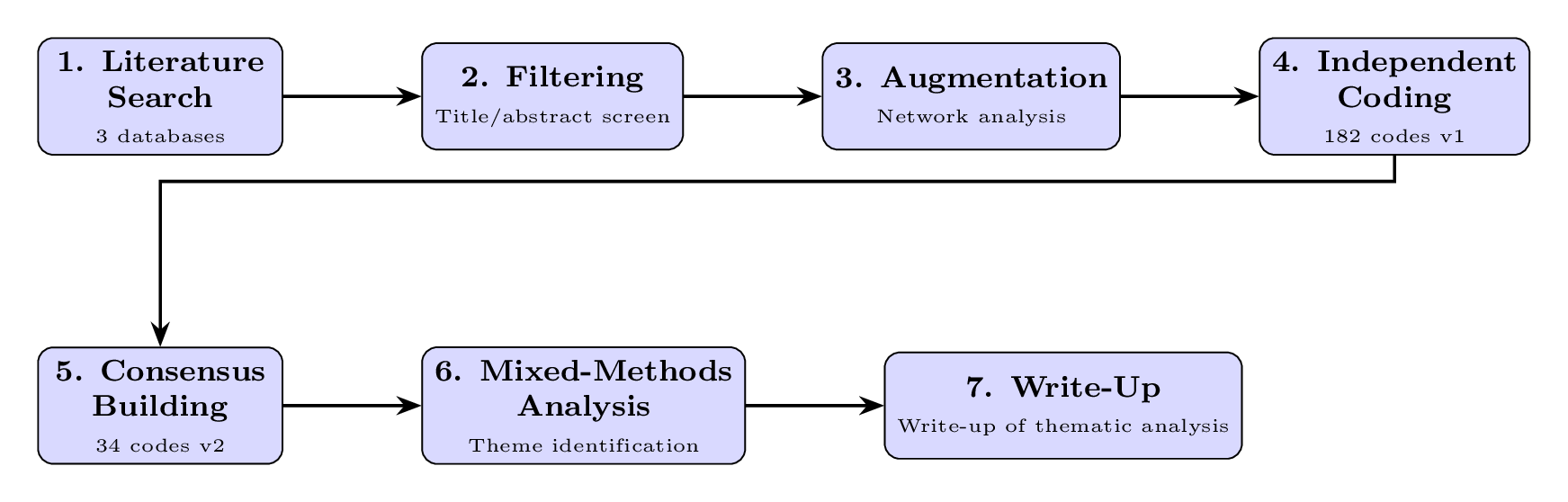


Figure 1: Seven-stage thematic review methodology pipeline

## Stage 1: Literature Search

A semi-systematic literature search was conducted across three major academic databases to ensure comprehensive coverage of the relevant literature.

### Search Strategy

The search was performed using the following databases:

- **IEEE Xplore**: Focus on engineering and computer science perspectives
- **PubMed**: Medical and healthcare literature
- **ScienceDirect**: Interdisciplinary coverage including social sciences

The search query used across all databases was:

TITLE-ABS-KEY: "digital twin*" AND (ethic* OR legal OR soci* OR policy)

### Search Results

The initial literature search yielded 29 papers that met our inclusion criteria. Table [1](#tab:search-results-supp) presents the complete list of papers identified through this systematic search, along with the database source from which each paper was retrieved (to be completed).

Table 1. Papers identified through literature search (n=29)

| Author | Title | Source |
| --- | --- | --- |
| Author | Title | Source |
| Armeni et al. (2022) | Digital Twins in Healthcare: Is It the Beginning of a New Era of Evidence-Based Medicine? A Critical Review | Research Rabbit |
| Ashraf et al. (2024) | Digital Twin for Neurology: An Introduction to a New Frontier in Healthcare | IEEE |
| Barricelli et al. (2019) | A Survey on Digital Twin: Definitions, Characteristics, Applications, and Design Implications | Research Rabbit |
| Begishev et al. (2024) | Digital Twins in Healthcare System: Communication between Society and Law | IEEE |
| Braun (2021) | Represent Me: Please! Towards an Ethics of Digital Twins in Medicine | Research Rabbit |
| Braun & Krutzinna (2022) | Digital Twins and the Ethics of Health Decision-Making Concerning Children | ScienceDirect |
| Bruynseels et al. (2018) | Digital Twins in Health Care: Ethical Implications of an Emerging Engineering Paradigm | PubMed |
| Chang et al. (2023) | Understanding the Need for Digital Twins’ Data in Patient Advocacy and Forecasting Oncology | PubMed |
| Chase et al. (2023) | Digital Twins and Automation of Care in the Intensive Care Unit | IEEE |
| Coorey et al. (2022) | The Health Digital Twin to Tackle Cardiovascular Disease—a Review of an Emerging Interdisciplinary Field | PubMed |
| Croatti et al. (2020) | On the Integration of Agents and Digital Twins in Healthcare | Research Rabbit |
| Dahir et al. (2023) | Chapter 4 - Challenges of Digital Twin in Healthcare | ScienceDirect |
| de Kerckhove (2021) | The Personal Digital Twin, Ethical Considerations | PubMed |
| Drummond & Coulet (2022) | Technical, Ethical, Legal, and Societal Challenges With Digital Twin Systems for the Management of Chronic Diseases in Children and Young People | PubMed |
| Fuller et al. (2020) | Digital Twin: Enabling Technologies, Challenges and Open Research | Research Rabbit |
| Huang et al. (2022) | Ethical Issues of Digital Twins for Personalized Health Care Service: Preliminary Mapping Study | PubMed |
| Iqbal et al. (2022) | The Use and Ethics of Digital Twins in Medicine | PubMed |
| Kamel Boulos & Zhang (2021) | Digital Twins: From Personalised Medicine to Precision Public Health | PubMed |
| Lauer-Schmaltz et al. (2024) | ETHICA: Designing Human Digital Twins—a Systematic Review and Proposed Methodology | IEEE |
| Leo et al. (2022) | Health Technology Assessment for In Silico Medicine: Social, Ethical and Legal Aspects | PubMed |
| Li et al. (2024) | The Status Quo and Future Prospects of Digital Twins for Healthcare | ScienceDirect |
| Lupton (2021) | Language Matters: The ’digital Twin’ Metaphor in Health and Medicine | PubMed |
| Mohr et al. (2024) | Navigating Challenges and Opportunities in Multi-Omics Integration for Personalized Healthcare | PubMed |
| Popa et al. (2021) | The Use of Digital Twins in Healthcare: Socio-Ethical Benefits and Socio-Ethical Risks | Research Rabbit |
| Sharma et al. (2023) | Integration of AI, Digital Twin and Internet of Medical Things (IoMT) for Healthcare 5.0: A Bibliometric Analysis | IEEE |
| Shengli (2021) | Is Human Digital Twin Possible? | ScienceDirect |
| Vallée (2024) | Envisioning the Future of Personalized Medicine: Role and Realities of Digital Twins | PubMed |
| Winter & Chico (2023) | Using the Non-Adoption, Abandonment, Scale-Up, Spread, and Sustainability (NASSS) Framework to Identify Barriers and Facilitators for the Implementation of Digital Twins in Cardiovascular Medicine | PubMed |
| Zhang et al. (2024) | Concepts and Applications of Digital Twins in Healthcare and Medicine | PubMed |

### Search Parameters

- Time period: 2010 onwards (reflecting when the concept "digital twin" gained traction)
- Language: English only
- Publication types: All peer-reviewed articles, conference papers, and reviews

The initial search across the three databases yielded a total of 106 papers for further filtering and review.

## Stage 2: Filtering

An initial filtering process was conducted based on title and abstract screening to remove papers that were clearly not relevant to our research questions.

### Search and Filtering Results

The initial search yielded 106 papers, which were then filtered based on title and abstract review. Papers were included if they:

- Explicitly discussed digital twins in healthcare contexts
- Addressed at least one ethical, legal, social, or implementation barrier issue
- Provided substantive analysis rather than purely technical descriptions
- Were accessible for full-text review

Papers excluded at this stage typically focused purely on technical implementations without ELSI considerations, or discussed digital twins in non-healthcare domains.

## Stage 3: Augmentation

To address potential gaps in the initial database search, we employed network analysis techniques using ResearchRabbit, a tool that identifies relevant papers through citation networks and semantic similarity.

This augmentation stage was crucial for identifying papers that used different terminology or were published in venues not covered by our initial search strategy, ensuring broader coverage of the ELSI literature.

### Network Analysis Process

- Input: The 23 papers identified from Stage 2 filtering
- Method: Citation network expansion and semantic similarity analysis
- Additional papers identified: 6 relevant papers not captured by initial search
- Final augmented dataset: 29 papers

### Final Filtering

A final filtering stage was set for this stage, but following a general review of the papers it was decided that no further exclusions would be made, resulting in a final set of 29 papers for detailed coding and analysis (see Table [1](#tab:search-results-supp)).

Table 2. Summary of literature search results by database

| **Database** | **Initial Results** | **After Title/Abstract Filtering** |
| --- | --- | --- |
| IEEE Xplore | 52 | 5 |
| PubMed | 23 | 14 |
| ScienceDirect | 31 | 4 |
| ResearchRabbit | 50 | 6 |
| **Total** | **156** | **29** |

## Stage 4: Independent Coding

Three of the co-authors (CB, PW, SQ) conducted detailed thematic coding of the 29 unique papers using an emergent coding approach.

### Coding Framework

Four main code categories were established prior to any review, and were pre-populated with 10 codes that we expected to see in the literature (e.g. data bias, data privacy and protection):

- **Ethical Issues**: Moral and value-based considerations
- **Legal Issues**: Regulatory and legal framework challenges
- **Social Issues**: Societal impacts and implications
- **Barriers**: Obstacles to practical implementation of digital twins, with an emphasis on realising the opportunities or reducing the risks associated with the above ethical, legal, and social issues.

### Coding Process

- Specific portions of the texts were annotated and assigned one of the existing codes or a new code if the coder deemed the annotation sufficiently different.
- Our preliminary codebooks comprised 182 distinct codes across all four categories
- These annotations and codes were extracted from the PDFs in a plain text format to support the next two stages (see [2.6](#subsec:supp-stage6))

## Stage 5: Consensus Formation

Following independent coding, the three coders engaged in iterative consensus-building discussions to refine and consolidate the initial codebook.

The consensus process resulted in a reduction from 182 codes (v1) to 34 codes (v2), across the following categories:

- Ethical Issues: 10 codes
- Social Issues: 9 codes
- Legal Issues: 6 codes
- Barriers: 9 codes

During this stage we carried out some initial quantitative analysis to help inform the consensus process. Specifically, we analysed the distribution of the 182 initial codes to understand which issues were most commonly discussed across the literature.

### Code Frequency Analysis

Figure [2](#fig:code-frequencies-v1) shows the distribution of code frequencies from the initial coding phase^[[1]](#footnote-1)^. The analysis revealed a highly skewed distribution, with a small number of codes appearing frequently across papers while many codes appeared only once or twice.


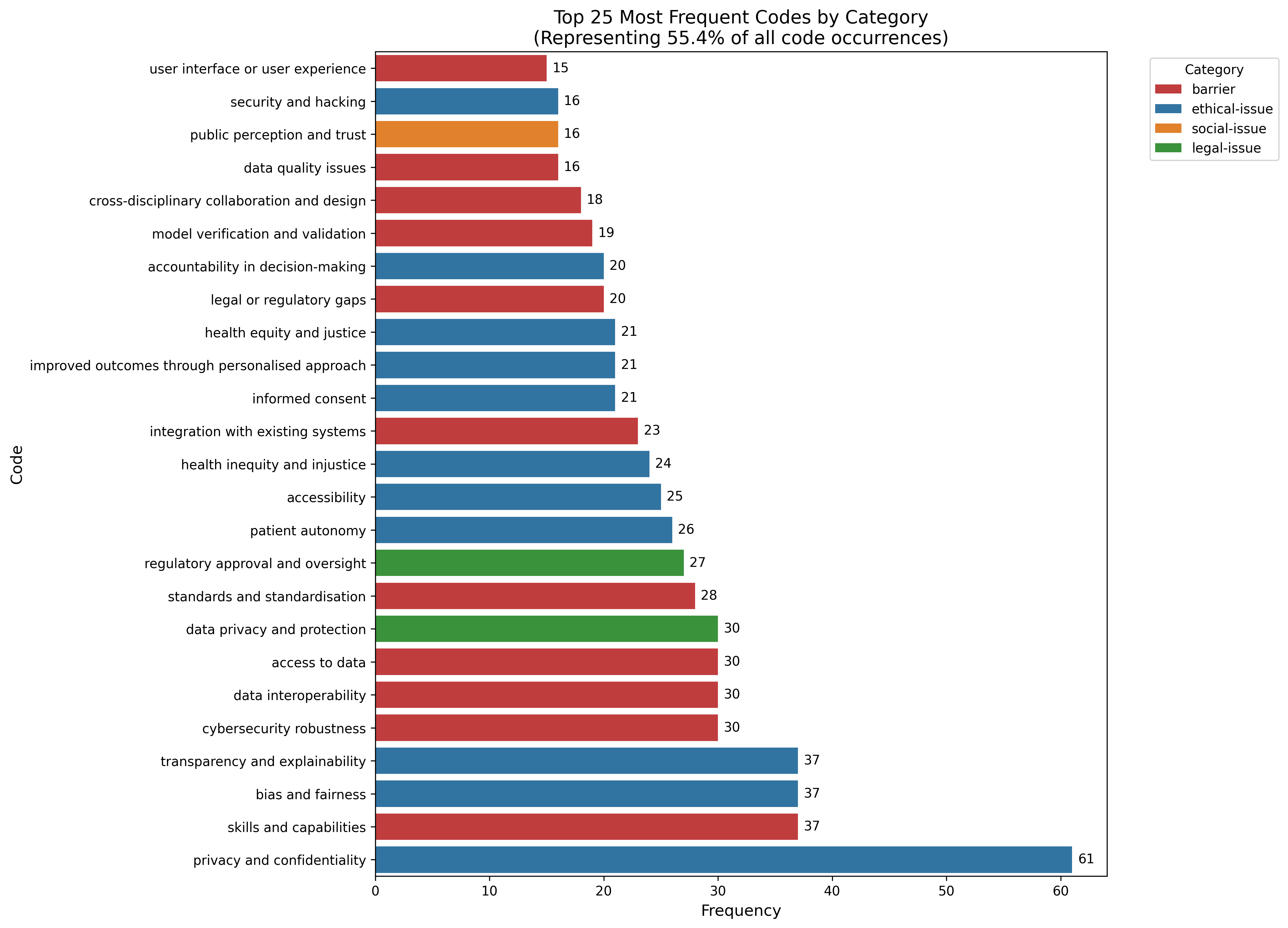


Figure 2: Frequency distribution of the 182 initial codes (version 1) across all 29 papers, organised by category (ethical issues, social issues, legal issues, and barriers).

We also conducted a Pareto analysis to examine the concentration of code usage (Figure [3](#fig:pareto-v1)). Interestingly, the analysis did not reveal the typical 80/20 distribution often seen in such analyses. Instead, we found that approximately 50% of all code occurrences came from the top 20% of codes, suggesting a more distributed discussion of issues across the literature rather than concentration on a few dominant themes. We report this finding for transparency, as it influenced our decision to maintain a relatively broad set of consolidated codes rather than focusing only on the most frequent ones.


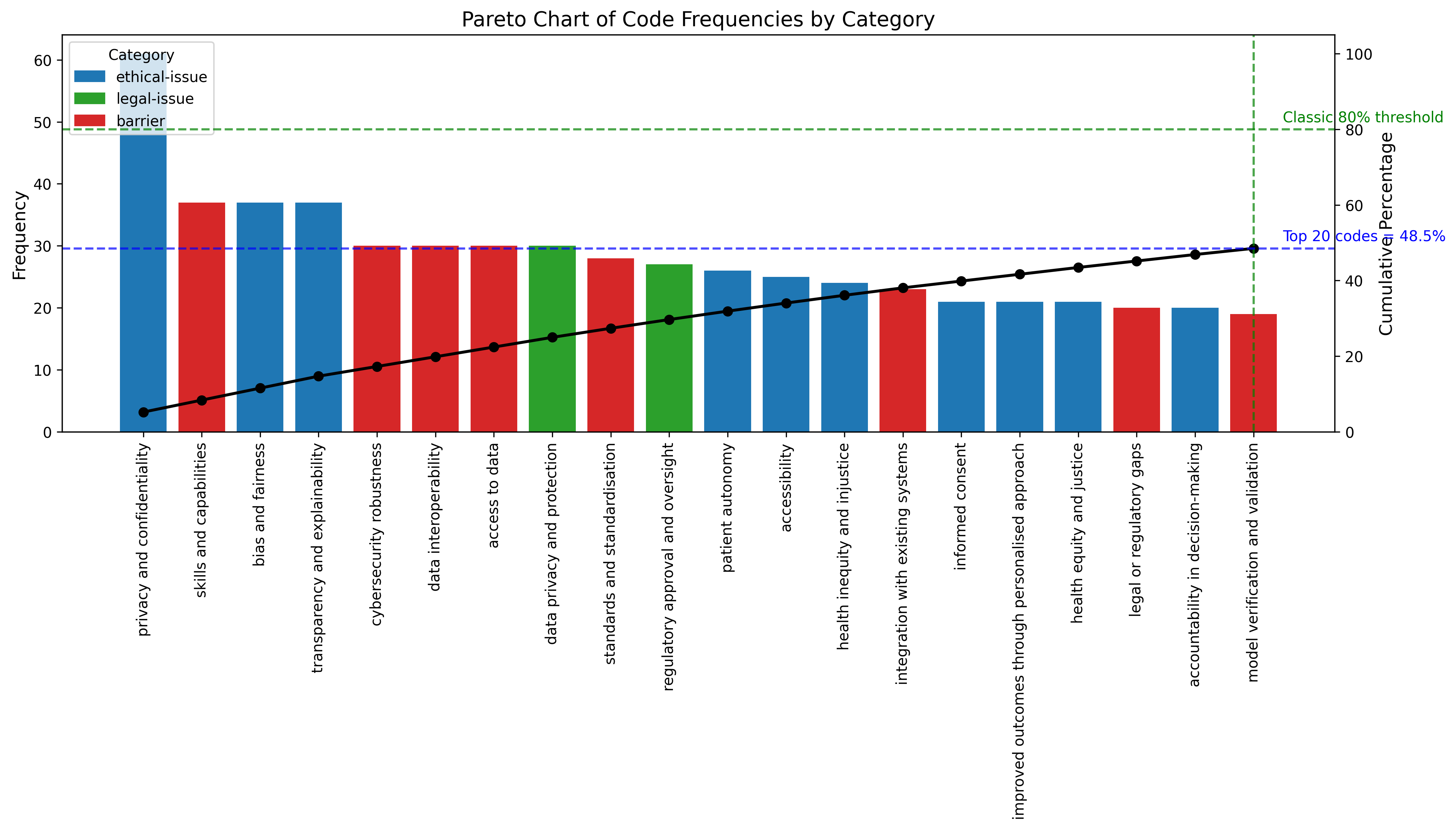


Figure 3: Pareto analysis of code frequencies showing the cumulative percentage of code occurrences. The analysis did not show the typical 80/20 distribution, indicating a more even spread of issues discussed in the literature.

While this initial consensus formation and analysis was sufficient to help us consolidate the codebook, a more in-depth analysis was then carried out in order to help define the major and minor themes (i.e. convert the v2 codes into substantive themes).

## Stage 6: Mixed-Methods Analysis

Following the consolidation of codes, we employed a comprehensive mixed-methods approach to identify major and minor themes. This analysis combined quantitative metrics with qualitative insights to ensure robust theme classification.

### Paper Spread Analysis

To distinguish between themes mentioned frequently within single papers versus those discussed broadly across the literature, we conducted a paper spread analysis. This compared:

- Raw frequency: Total mentions across all papers (including multiple uses of the same code within a single paper)
- Paper spread: Number of unique papers mentioning each theme

The analysis revealed that data-challenges dominated both measures (107 mentions across 25 papers), while some codes like technical-infrastructure-scalability showed high frequency (54 mentions) but narrower spread (13 papers), suggesting more concentrated discussion.

Figure [4](#fig:frequency-vs-spread) illustrates this relationship between raw frequency and paper spread, clearly showing data-challenges in the top-right corner with both high frequency and high spread. Figure [5](#fig:top-themes-freq-spread) provides an alternative view, comparing the top 15 codes by their total frequency (blue bars) against their paper spread (orange bars).


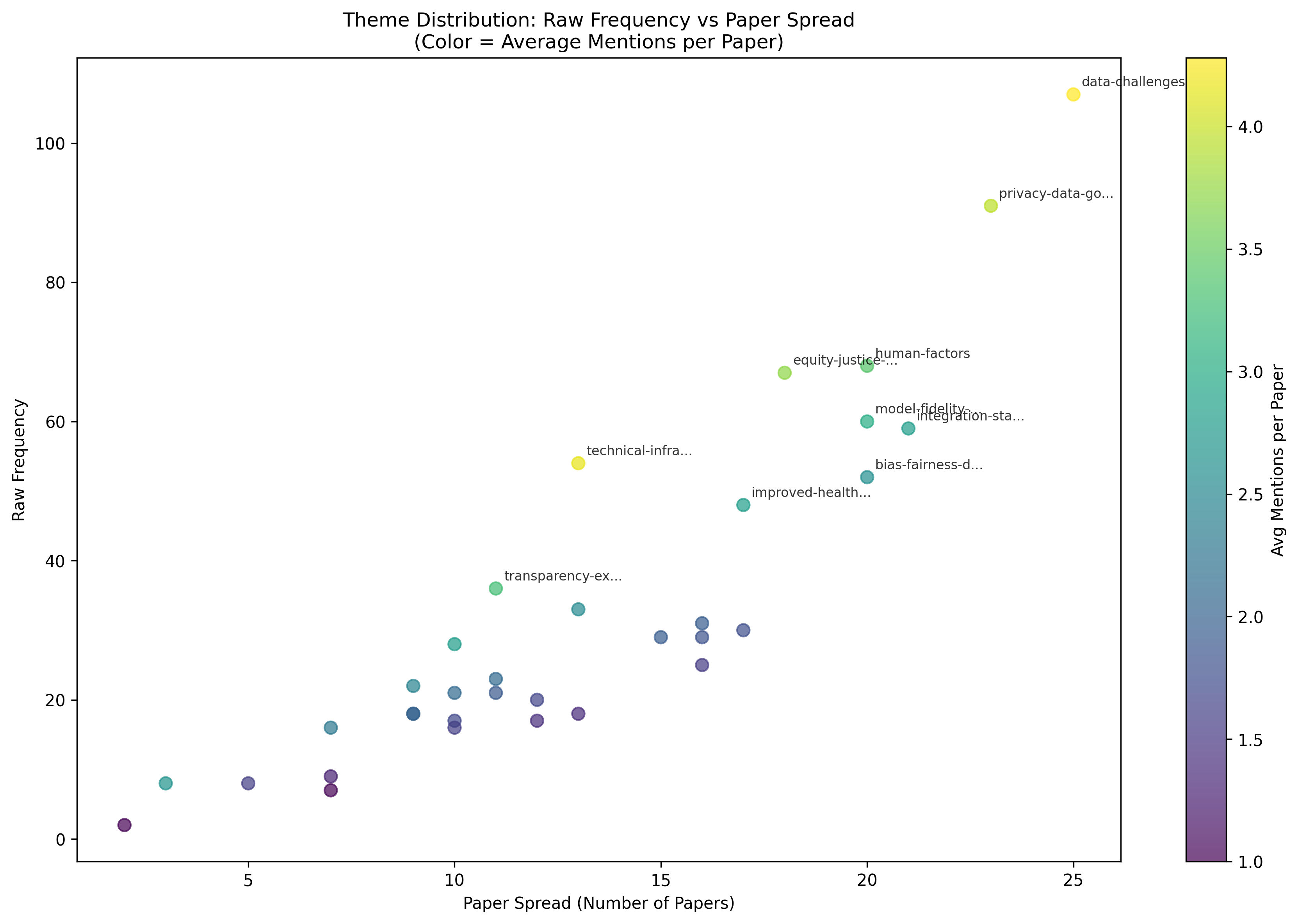


Figure 4: Scatter plot showing raw frequency (y-axis) vs. paper spread (x-axis) for all themes. The position of data-challenges in the top-right demonstrates its dominance on both measures.


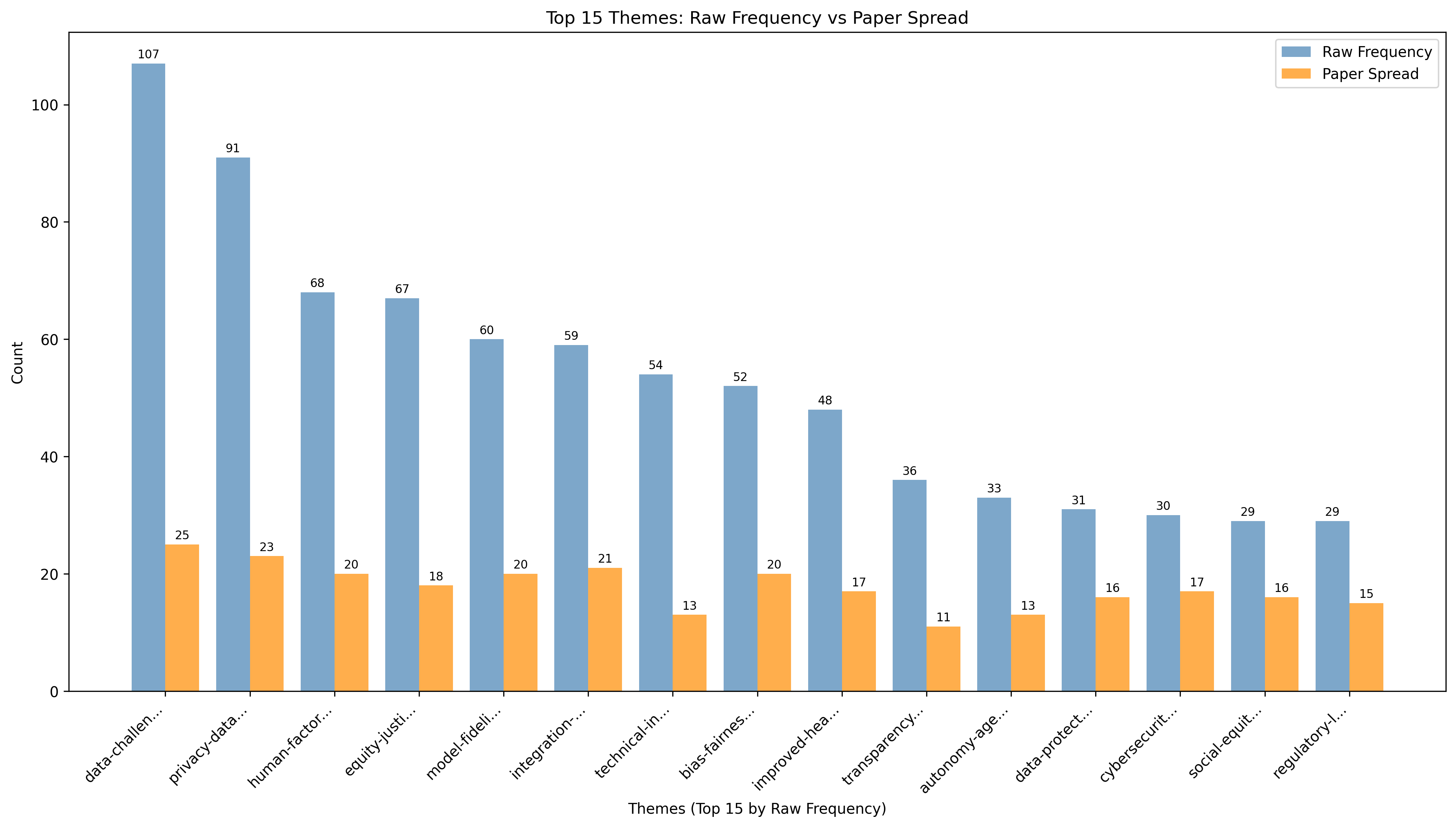


Figure 5: Comparison of the top 15 themes by total frequency and paper spread, revealing which themes are discussed widely versus intensively.

### Co-occurrence Analysis

We analysed which codes appeared together within the same papers to identify thematic relationships. The strongest relationship emerged between data-challenges and privacy-data-governance (co-occurring in 22 papers). Seven codes demonstrated maximum connectivity, appearing with every other code across the literature: autonomy-agency, bias-fairness-discrimination, equity-justice-accessibility, human-factors, improved-health-outcomes, model-fidelity-validation, and regulatory-legal-gaps.

Figure [6](#fig:cooccurrence-heatmap) presents a detailed heatmap showing which specific codes co-occur most frequently, with darker colours indicating stronger relationships. Figure [7](#fig:cooccurrence-network) provides a network visualisation of the top 15 most connected codes, revealing cross-category relationships and the strongest conceptual connections.


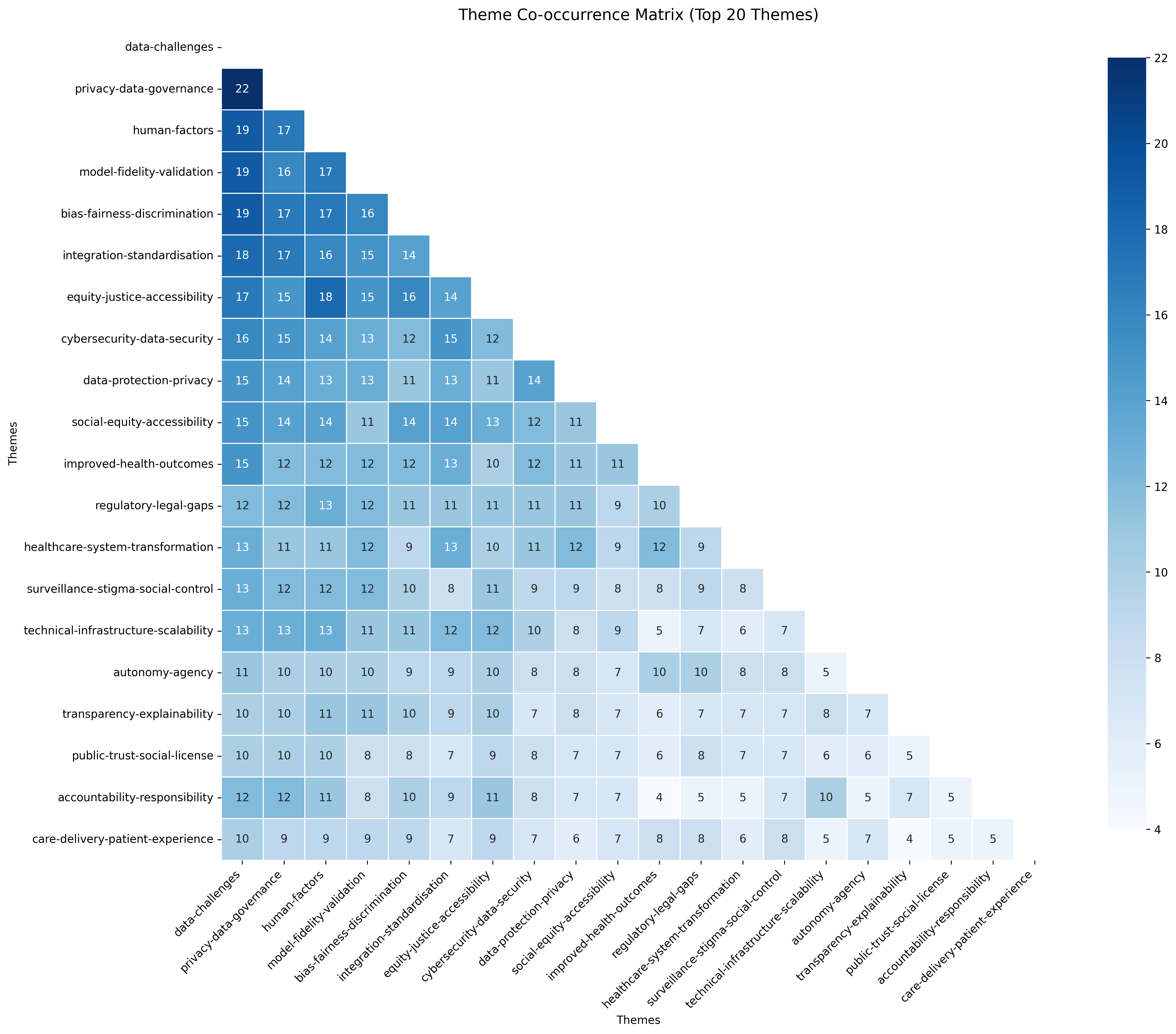


Figure 6: Co-occurrence heatmap showing the frequency with which pairs of codes appear together in the same papers. Darker colours indicate stronger relationships.


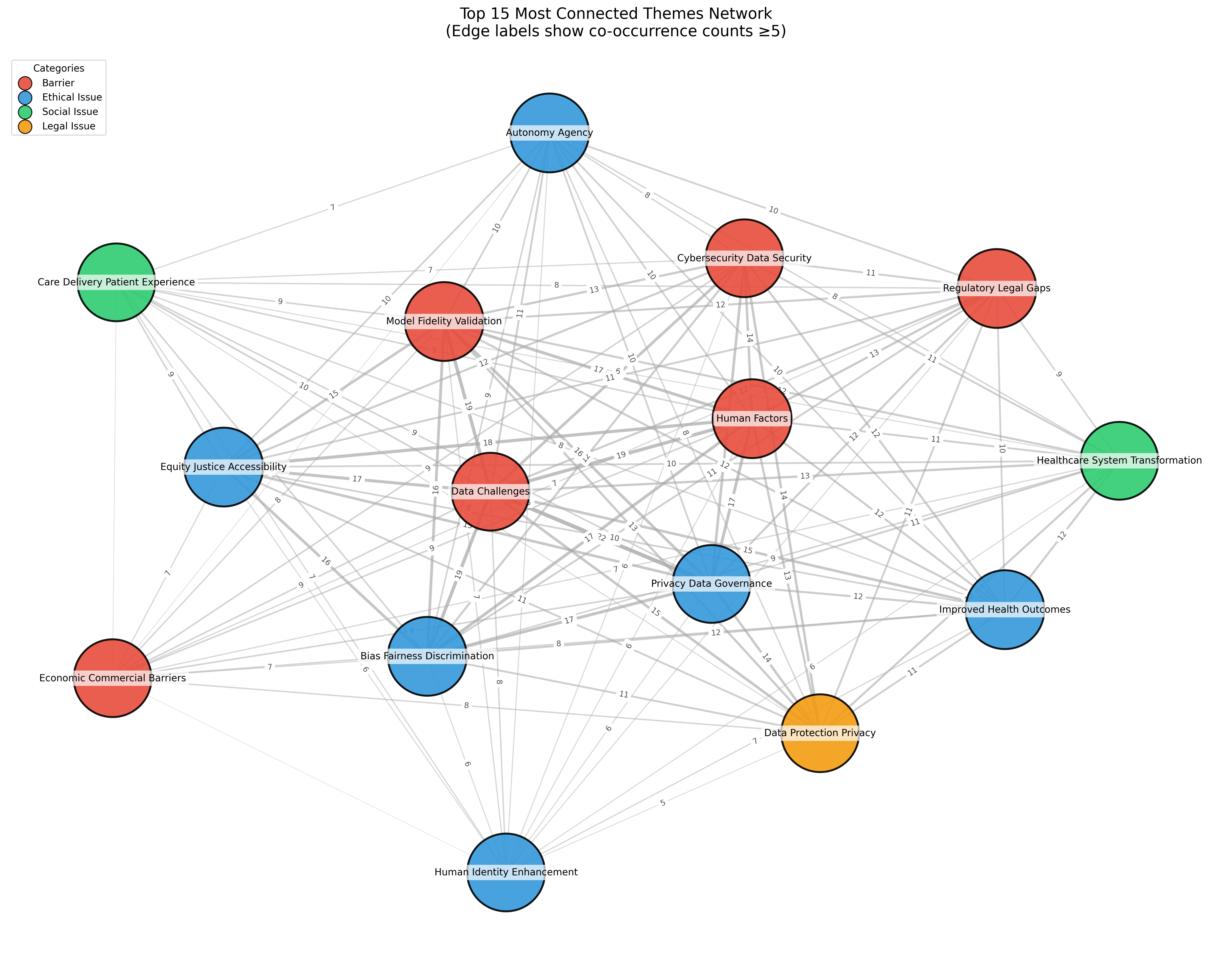


Figure 7: Network visualisation showing the top 15 most connected codes. Edge labels indicate the number of papers in which theme pairs co-occur, revealing strong cross-category conceptual connections.

### Composite Scoring

Rather than relying solely on frequency to define our major and minor themes, we developed a weighted composite score combining:

- Frequency (45% weight): Raw importance based on total mentions
- Paper spread (30% weight): Breadth of discussion across the corpus
- Network centrality (25% weight): Conceptual connectivity to other codes

This approach treated all categories equally, allowing natural patterns to emerge without structural advantages for any particular concern type.

### Statistical Threshold Identification

With no obvious natural breakpoint in the composite score distribution, we applied three complementary statistical methods:

- **Elbow Method (40% weight)**: Identifies where additional clusters provide diminishing returns
- **Gap Statistic (30% weight)**: Compares clustering quality against null distributions
- **Silhouette Analysis (30% weight)**: Measures cluster separation and internal cohesion

Whilst each method suggested different optimal cluster numbers (Elbow: 2, Gap: 1, Silhouette: 7), our research objective required binary classification (i.e. major and minor themes). We therefore standardised all calculations using 2-cluster solutions, with higher weighting for the Elbow Method as it naturally aligned with our binary classification goal. The ensemble approach yielded a threshold of 0.433, identifying 11 globally major themes:

1. data-challenges (0.883) - barrier
2. privacy-data-governance (0.788) - ethical issue
3. human-factors (0.655) - barrier
4. equity-justice-accessibility (0.624) - ethical issue
5. integration-standardisation (0.621) - barrier
6. model-fidelity-validation (0.620) - barrier
7. bias-fairness-discrimination (0.586) - ethical issue
8. improved-health-outcomes (0.530) - ethical issue
9. technical-infrastructure-scalability (0.495) - barrier
10. cybersecurity-data-security (0.448) - barrier
11. data-protection-privacy (0.440) - legal issue

Figure [8](#fig:composite-scores) shows the top 20 codes ranked by their composite scores, whilst Figure [9](#fig:robust-threshold) illustrates the statistical threshold identification process and Figure [10](#fig:threshold-comparison) compares the thresholds derived from each method.


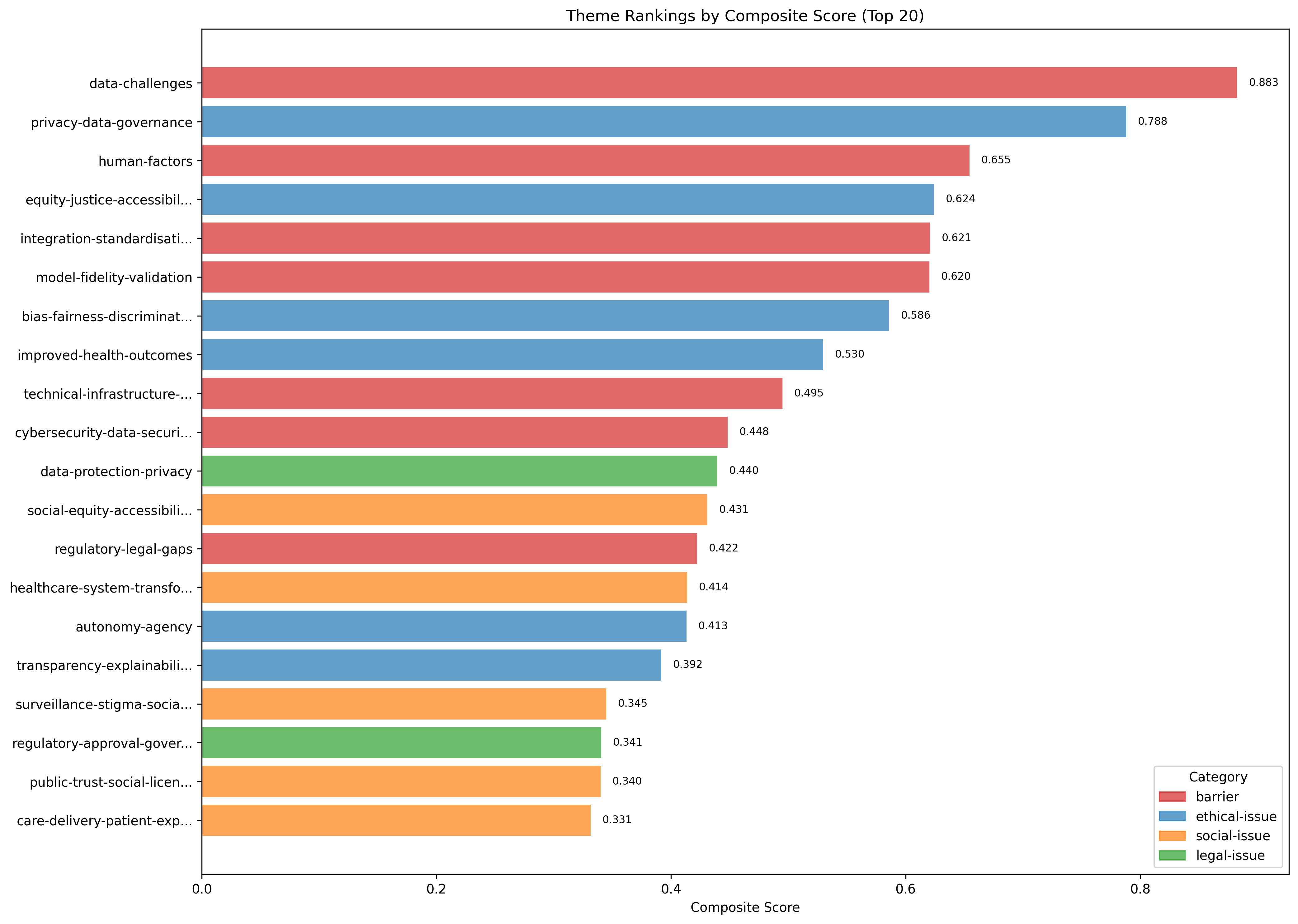


Figure 8: Top 20 themes ranked by composite scores, combining frequency, paper spread, and network centrality measures.


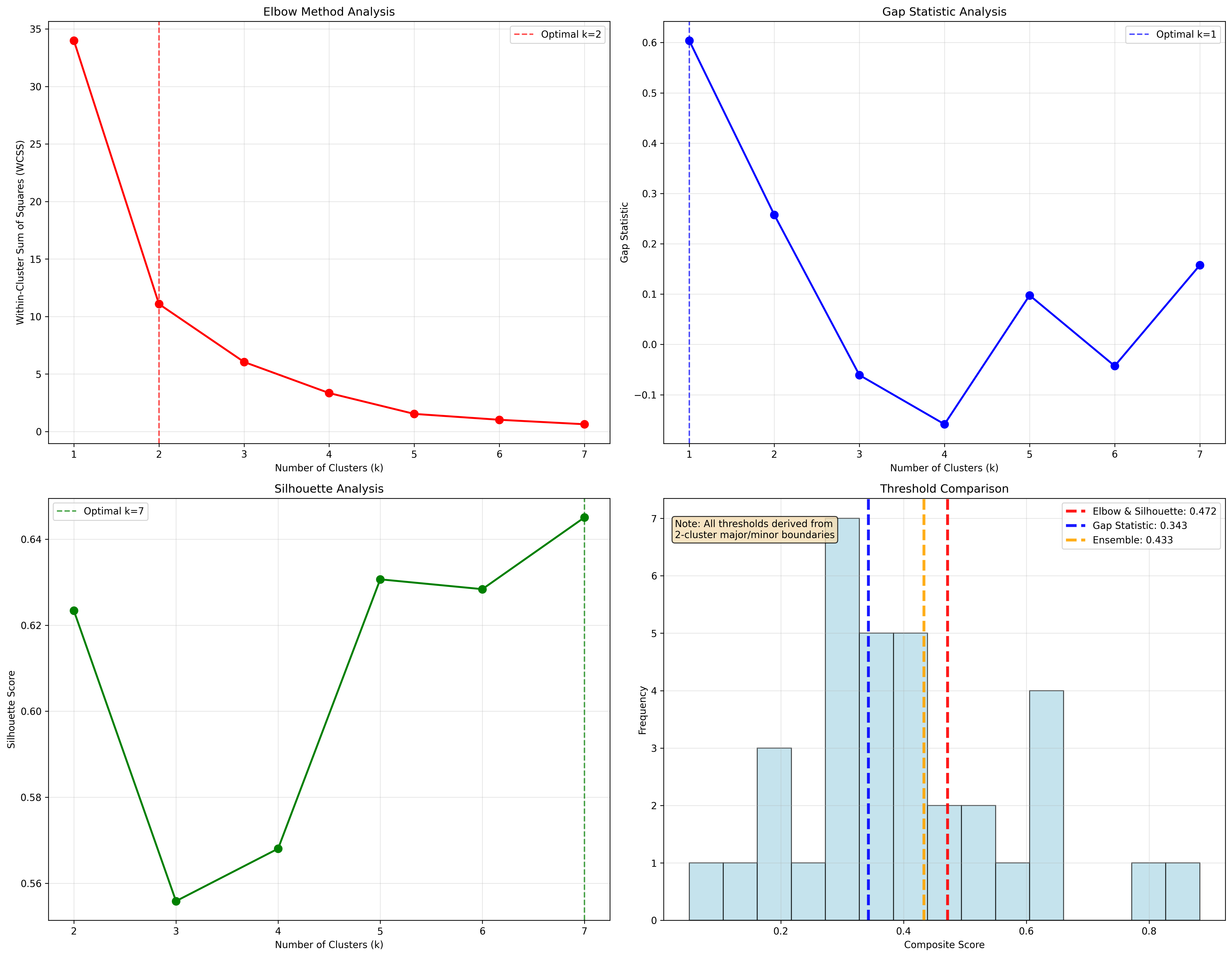


Figure 9: Statistical threshold analysis showing the application of three clustering methods (Elbow Method, Gap Statistic, and Silhouette Analysis) to identify the major/minor theme boundary.


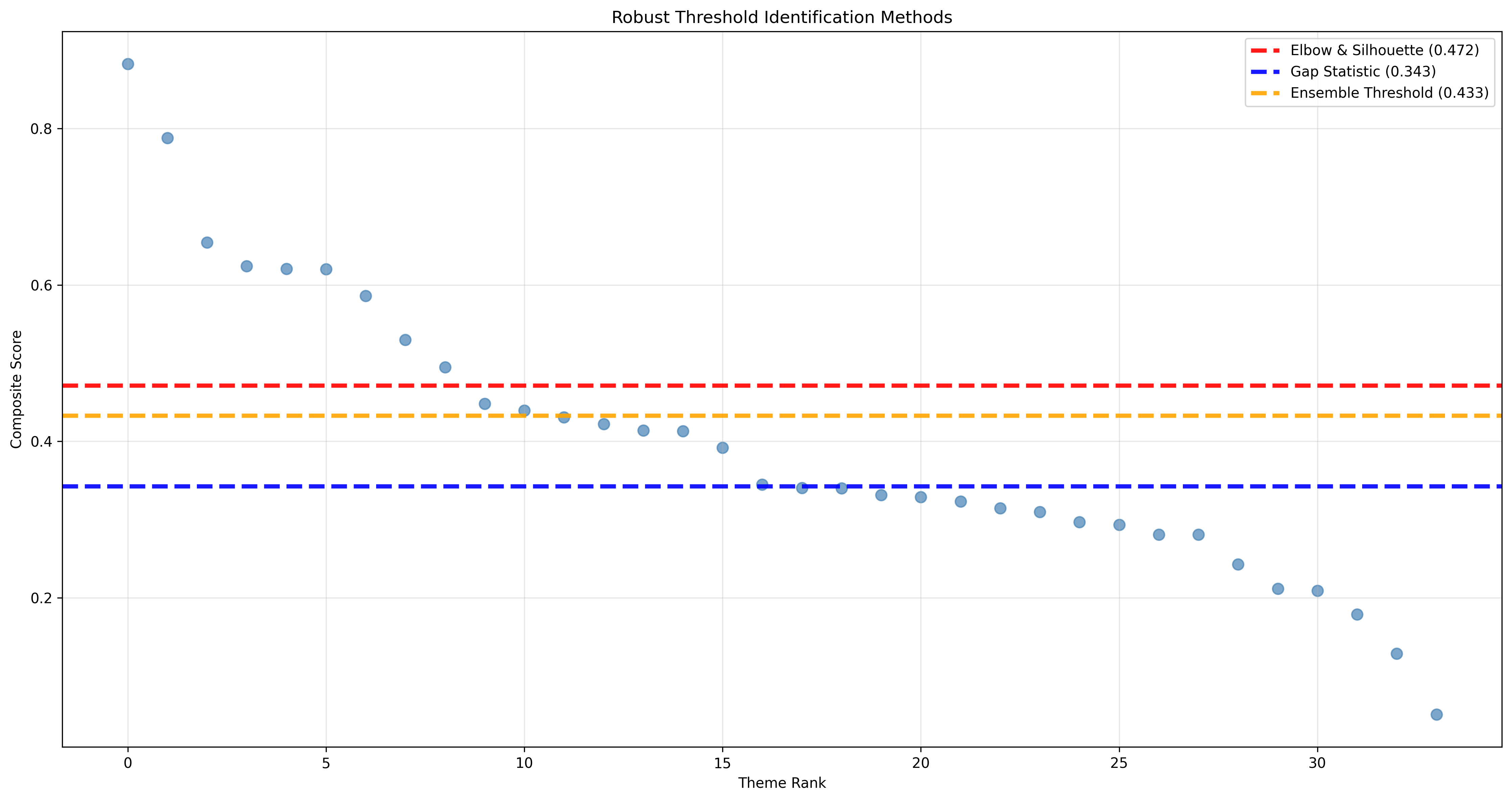


Figure 10: Comparison of thresholds derived from different statistical methods, showing convergence between Elbow Method and Silhouette Analysis at 0.472.

### Category-Specific Analysis

The global analysis revealed systematic underrepresentation of social and legal issues, with only one legal theme and no social themes reaching global major status. To address this, we applied the same ensemble approach *within* each category:

- **Barriers**: 4 of 9 themes classified as major (threshold: 0.558)
- **Ethical Issues**: 4 of 10 themes classified as major (threshold: 0.471)
- **Social Issues**: 8 of 9 themes classified as major (threshold: 0.147)^[[2]](#footnote-2)^
- **Legal Issues**: 3 of 6 themes classified as major (threshold: 0.252)

This category-specific analysis helped reveal 10 additional (category-specific) major themes that would have been overlooked by global analysis alone. Figure [11](#fig:category-specific) visualises the major versus minor theme classifications within each category, revealing the hidden structure in social and legal domains that would have been missed by global analysis alone.


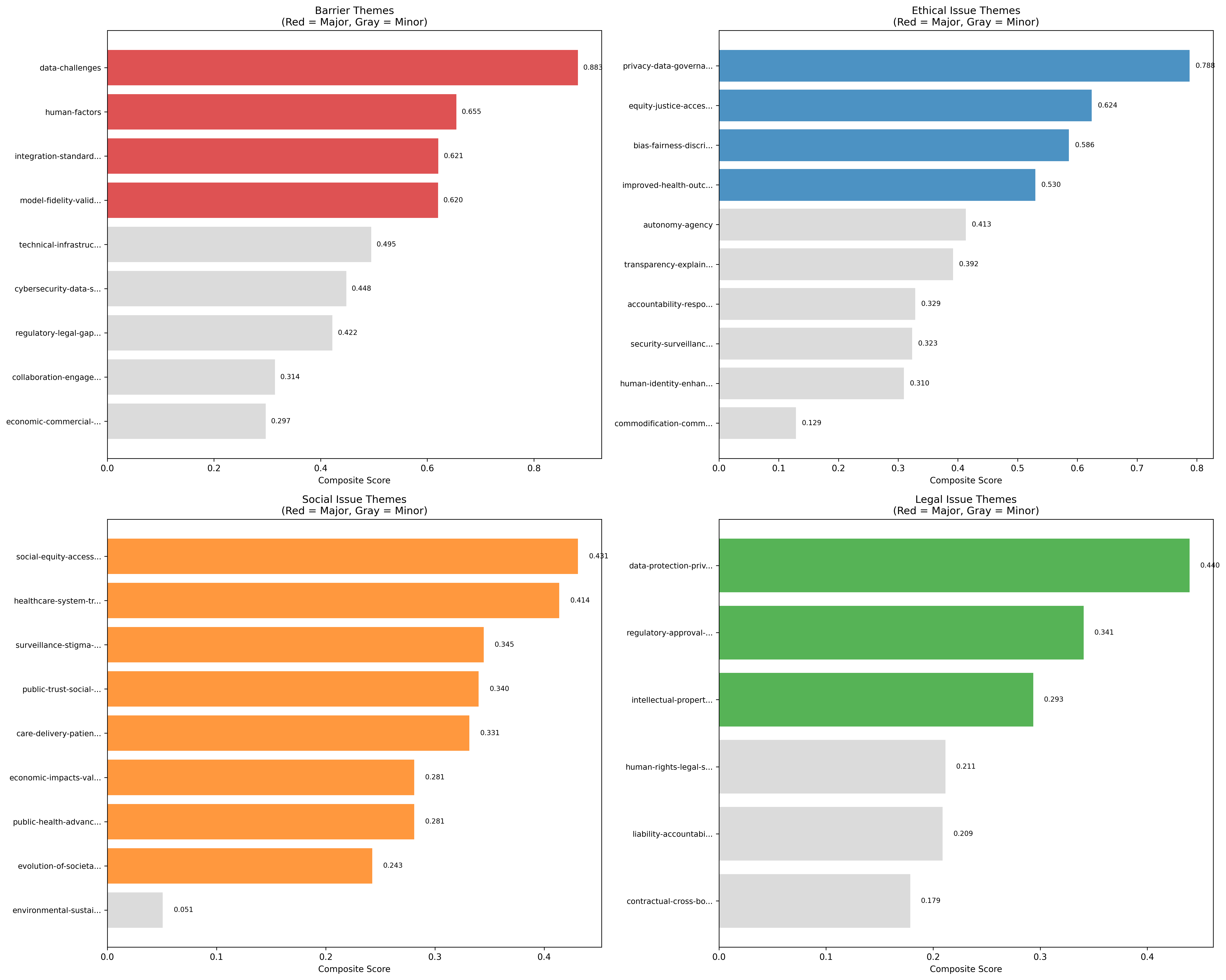


Figure 11: Category-specific theme classifications showing major (dark) versus minor (light) themes within each domain. Note the predominance of major themes within the social issues category.

### Barrier-Issue Co-occurrence Analysis

To understand how implementation barriers relate to ethical, legal, and social issues, we created a co-occurrence matrix examining barrier-issue pairs. The analysis revealed 223 unique relationships (of 225 possible), with 99.1% average connectivity.

The strongest relationships were:

- data-challenges $\leftrightarrow$ privacy-data-governance (22 papers, 95.7%)
- data-challenges $\leftrightarrow$ bias-fairness-discrimination (19 papers, 95.0%)
- human-factors $\leftrightarrow$ equity-justice-accessibility (18 papers, 100%)
- human-factors $\leftrightarrow$ privacy-data-governance (17 papers, 73.9%)
- integration-standardisation $\leftrightarrow$ privacy-data-governance (17 papers, 73.9%)

Figure [12](#fig:barrier-issue-heatmap) presents the complete barrier-issue co-occurrence matrix, revealing the strength of relationships between implementation challenges and ELSI concerns.


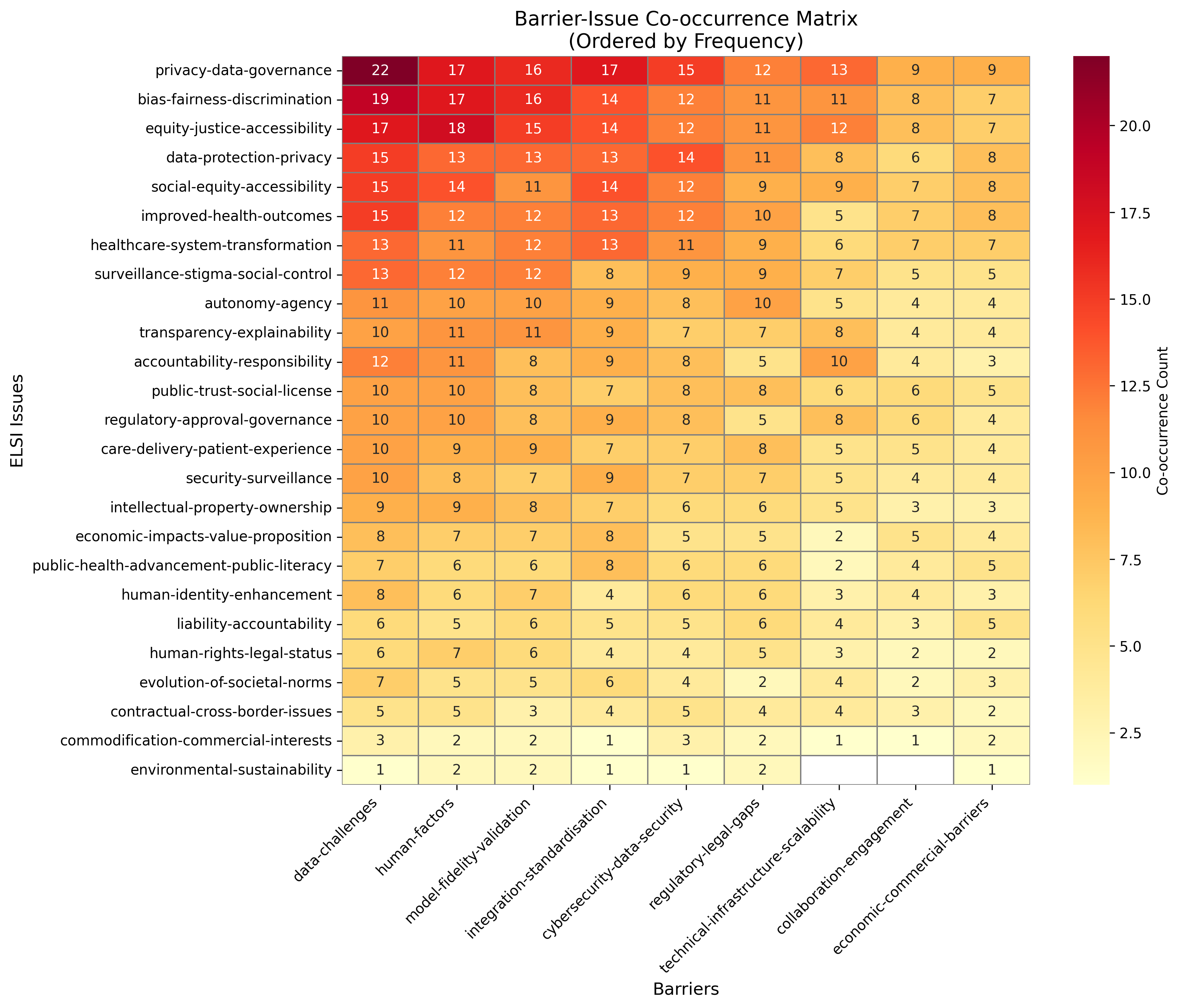


Figure 12: Barrier-issue co-occurrence heatmap showing the frequency with which implementation barriers appear alongside ethical, legal, and social issues. Darker colours indicate stronger relationships, with data challenges showing particularly strong connections to privacy and bias concerns.

This was a surprisingly high level of connectivity, and could be taken to demonstrate that the literature takes an integrated and holistic view of digital twin challenges, and recognises deep interconnections between technical barriers and ELSI concerns.

However, as we note in the limitations, an alternative interpretation is that the search engines we used impart a selection bias towards technical and practical discussions, rendering mention of ethical, legal, and social issues as (at times) fairly superficial, and thus easy to just mention but with little in-depth discussion.

Following the quantitative analysis, we were then able to define our updated major and minor themes from the v2 codes. This required a) revisiting the original sources to understand the original context for the annotation, and b) evaluating the full set of coded annotations (e.g. all uses of autonomy-agency) to help develop a theme that represented the literature as a whole.

## Stage 7: Analysis and Write-Up

The full write-up and thematic analysis is presented in the main paper. Here, we present a summary view of the major and minor themes. Each theme is followed by a tag that indicates whether it was labelled as major or minor in the global (G) and category-specific (CS) rankings.

### Ethical Issues

1. **Privacy & Data Governance (G: Major / CS: Major):** Addresses broader ethical concerns related to the collection, use, sharing, control, and governance of sensitive health data within DT systems. Includes informed consent models, data ownership debates, de-identification risks, potential misuse beyond legal breaches, and fairness in data governance practices.
2. **Equity, Justice & Accessibility (G: Major / CS: Major):** Focuses on the fair distribution of DT benefits and risks as a matter of justice and rights. Addresses potential disparities in access based on cost, digital literacy, geography, or socioeconomic status, and ensuring social inclusion from an ethical standpoint.
3. **Bias, Fairness & Discrimination (G: Major / CS: Major):** Examines the potential for DTs to perpetuate or exacerbate existing biases (e.g., racial, gender, socioeconomic) due to unrepresentative data or flawed algorithms, leading to unfair or discriminatory outcomes. Includes issues related to patient heterogeneity.
4. **Improved Health Outcomes (G: Major / CS: Major):** Considers the core ethical duty to act in the best interest of the patient, maximising potential benefits (like improved/earlier diagnosis, personalised treatment, reduced trial risks) whilst minimising harm (including overdiagnosis or unnecessary interventions)—such as beneficence and non-maleficence in biomedical ethics. Covers predictive maintenance aspects related to patient safety.
5. **Autonomy & Agency (G: Minor / CS: Minor):** Concerns the capacity of both patients and clinicians to make independent, informed decisions regarding healthcare, considering potential over-reliance on DTs, deskilling, medical paternalism, and the right *not* to know certain health information.
6. **Transparency & Explainability (G: Minor / CS: Minor):** Highlights the need for clarity in how DTs function, how decisions are made, how interpretable a model or system is, and how data is used, ensuring that both clinicians and patients can understand and trust the technology (e.g. ’black box’ problem).
7. **Accountability & Responsibility (G: Minor / CS: Minor):** Deals with determining who is liable when errors or adverse outcomes occur due to DT malfunction or misuse, particularly in complex decision-making scenarios.
8. **Security & Surveillance (G: Minor / CS: Minor):** Focuses on the ethical risks of unauthorised access, hacking, data breaches, and the potential for DTs to be used for intrusive surveillance or behaviour manipulation, distinct from societal impacts.
9. **Human Identity & Enhancement (G: Minor / CS: Minor):** Explores how DTs might alter human capabilities beyond therapy, impacting concepts of normality, identity, and the human condition. Also covers psychological impacts (on patients and also healthcare professionals), cognitive effects, social relatedness, representation, and digital legacy/afterlife.
10. **Commodification & Commercial Interests (G: Minor / CS: Minor):** Addresses the ethical implications of treating patient data as a commodity, potential exploitation for profit, monetisation without patient benefit, and the risk of corporate interests overriding patient well-being.

### Legal Issues

1. **Data Protection & Privacy (G: Major / CS: Major):** Covers specific legal requirements for safeguarding personal health data, including compliance with regulations like GDPR, data breach notification rules, anonymisation standards, and mechanisms for legal enforcement.
2. **Regulatory Approval & Governance (G: Minor / CS: Major):** Addresses the legal frameworks for validating, approving, and overseeing DTs as medical technologies, including classification, pre-market approval, standardisation, and ongoing compliance monitoring.
3. **Human Rights & Legal Status (G: Minor / CS: Minor):** Explores the intersection of DTs with fundamental human rights, including non-discrimination, bodily integrity, the right to consent (or refuse), and the evolving legal definition or status of DTs themselves.
4. **Intellectual Property & Ownership (G: Minor / CS: Major):** Deals with legal rights concerning the ownership of the DT itself, the data it uses/generates, and any innovations derived from it, including patents and licensing. Importantly, this can also include bounds on acceptable use of digital twin, as determined by informed consent, and means for redress by affected person.
5. **Liability & Accountability (G: Minor / CS: Minor):** Concerns the allocation of legal responsibility and accountability when digital twins cause harm or errors, including issues of manufacturer vs. clinician liability and mechanisms for redress or compensation.
6. **Contractual & Cross-Border Issues (G: Minor / CS: Minor):** Focuses on legal agreements governing data use, licensing, sharing, and transfer, particularly across different jurisdictions, including smart contracts and challenges related to international regulations.

### Social Issues

1. **Social Equity & Accessibility (G: Minor / CS: Major):** Addresses practical disparities in access to DT technology and its benefits, considering the digital divide, socioeconomic barriers, patient diversity, global health justice implications, and the role of capabilities like remote monitoring in bridging gaps.
2. **Healthcare System Transformation (G: Minor / CS: Major):** Considers the broader impacts on the healthcare system, including workforce changes (job displacement, de-skilling, training needs), process optimisation (e.g., hospital management, patient management), enhanced safety/efficiency, policy implications, and the use of DTs for medical devices/systems rather than solely patients.
3. **Surveillance, Stigma & Social Control (G: Minor / CS: Major):** Addresses the societal impacts and public concerns related to increased surveillance facilitated by DTs (by state, corporations, or individuals), potential for social stigmatisation based on DT data, the spread/impact of health misinformation via DTs, and issues of oversight and control within society.
4. **Public Trust & Social License (G: Minor / CS: Major):** Focuses on societal perceptions, including trust (or mistrust) in DT technology and its outputs, issues related to adoption (including unrealistic expectations), and patient/society acceptance of DT technologies.
5. **Care Delivery & Patient Experience (G: Minor / CS: Major):** Examines how DTs impact the nature of healthcare interactions, including the doctor-patient relationship, potential dehumanisation, disruptions to daily life, usability, convenience, over/under-reliance, diverted focus, and the concept of a digital afterlife.
6. **Economic Impacts & Value Proposition (G: Minor / CS: Major):** Examines the financial implications of DTs, including potential cost savings (e.g., lower healthcare/trial costs, affordability), increased costs (development, implementation), the overall value proposition, and driving infrastructure development.
7. **Public Health Advancement & Public Literacy (G: Minor / CS: Major):** Highlights the potential of DTs to improve public health outcomes through better research, simulation (’what-if’ scenarios), patient stratification, understanding disease burdens, long-term health management, knowledge generation, and applications like healthier urban planning. But, dependent on the perceived importance of digital/health literacy for patients and the public (e.g. PPIE and community engagement).
8. **Evolution of Societal Norms (G: Minor / CS: Major):** Explores the need for societal and cultural sensitivity in DT design and deployment, potential impact and shaping of existing social norms.
9. **Environmental Sustainability (G: Minor / CS: Minor):** Considers the environmental footprint of DT technology, including energy consumption for data storage/processing and the lifecycle impacts of associated hardware.

### Barriers

1. **Data Challenges (G: Major / CS: Major):** Difficulties in obtaining sufficient, high-quality, varied, and interoperable data; ensuring data provenance and managing heterogeneity. Overcoming requires standardised data formats, robust data cleaning/fusion techniques, secure data sharing platforms (e.g., using blockchain for provenance), and addressing ethical/legal constraints on data access.
2. **Human Factors (G: Major / CS: Major):** Lack of necessary skills and training among clinicians and patients; distrust in AI/DTs due to lack of transparency or insufficient accuracy and robustness, poor usability and interpretability, flawed assumptions, or unclear visualisation. Requires human-centred design approaches, improved explainability methods, targeted training programmes, and addressing user perceptions/biases.
3. **Integration & Standardisation (G: Major / CS: Major):** Challenges integrating DTs with existing healthcare IT systems (EHRs, etc.) and workflows, and the lack of common standards for data, models, communication protocols, and system architecture. Requires development and adoption of interoperability standards (e.g., via consortia), modular design, and clear system specifications.
4. **Model Fidelity & Validation (G: Major / CS: Major):** Ensuring model accuracy, reliability, reproducibility, and generalisability; validating models against reality; managing scientific/epistemic complexity, algorithm limitations (e.g., catastrophic forgetting), and bridging the simulation-reality gap. Requires rigorous validation methodologies, benchmark tests, more research into complex biological systems, and developing robust, adaptable algorithms.
5. **Technical Infrastructure & Scalability (G: Major / CS: Minor):** Limitations in computational power, sensor functionality, storage, network latency (especially for real-time processing), system scalability, device connectivity (e.g. IoT), and maintaining consistent operation (e.g., predictive maintenance). Solutions involve investing in high-performance computing, advanced networking, and developing scalable, modular architectures.
6. **Cybersecurity & Data Security (G: Major / CS: Minor):** Protecting sensitive health data and DT systems from unauthorised access, breaches, malware, and ensuring data integrity and confidentiality throughout the lifecycle. Requires robust security measures like encryption, access controls, vulnerability detection, secure coding practices, and potentially blockchain for immutable records.
7. **Regulatory & Legal Gaps (G: Minor / CS: Minor):** Absence of clear, specific, harmonised regulations and legal definitions for DT development, validation, approval, use, and addressing issues like distributed responsibility or opt-out mechanisms. Requires proactive engagement between developers, regulators, and legal experts to establish clear guidelines, approval pathways (potentially adapting existing ones), and address definitional ambiguity.
8. **Collaboration & Engagement (G: Minor / CS: Minor):** Difficulties fostering effective communication and collaboration between diverse stakeholders (e.g. clinicians, engineers, data scientists, patients, regulators, industry) and ensuring meaningful patient/public involvement and engagement (PPIE) due to differing backgrounds, goals, or lack of resources. Requires dedicated co-design processes, clear communication strategies, and adequate resources for PPIE.
9. **Economic & Commercial Barriers (G: Minor / CS: Minor):** High costs associated with development, implementation, infrastructure, and maintenance; difficulty justifying the value proposition and securing financial investment; potential conflicts arising from commercial interests influencing development or data use. Requires clear cost-benefit analyses, sustainable funding models, and governance structures to manage commercial influences.

# Minor Themes from Thematic Analysis

This section contains the detailed analysis of minor themes identified in our thematic review. While these themes received less attention in the reviewed literature than the major themes presented in the main manuscript, they represent important emerging concerns that may become increasingly significant as digital twin technologies mature and move from research to clinical practice.

## Minor Ethical Themes

Six minor ethical themes represent emerging concerns requiring monitoring as DT technologies mature: autonomy and agency, transparency and explainability, accountability and responsibility, security and surveillance, human identity and enhancement, and commodification and commercial interests. These received less attention in the reviewed literature yet may prove increasingly significant as DTs move from research to clinical practice.

The classification of ‘autonomy and agency’ as minor is striking given autonomy’s status as a foundational bioethical principle alongside beneficence, non-maleficence, and justice (Beauchamp and Childress 2013). This neglect may reflect framing DTs as clinical decision support tools enhancing professional judgment rather than patient empowerment tools. The technical complexity requiring sophisticated expertise may implicitly justify prioritising professional expertise over patient autonomy—a notable shift from traditional bioethical frameworks, suggesting DT implementations may be reconstructing physician-patient relationships around technological mediation rather than patient agency.

## Minor Legal Themes

The three minor legal themes—human rights and legal status, liability and accountability frameworks, and contractual and cross-border issues—represent regulatory frontier issues. There are several explanations for why these themes received limited attention in the existing literature. First, it may reflect the nascent state of DT legal scholarship and the tendency for legal analysis to lag behind technological development. Second, the scope of our literature search may have resulted in missed papers that either dealt with these themes directly or addressed them but in connection with data-driven technologies more generally (see “Supplementary Material”).

Human rights considerations were typically addressed at a high level of abstraction, though Braun (2021, 399) is a notable exception with their observation that DTs could “interfere with the bodily integrity of a person or pose challenges which cannot be addressed.” This concept of *bodily integrity*, which encompasses both physical and mental dimensions as recognised in Article 3 of the EU Charter of Fundamental Rights, remains under-explored in the DT literature. For instance, the effect of continuous health monitoring and predictive health modelling on both bodily and mental integrity presents both conceptual and empirical questions that warrant further investigation.

Contractual issues become particularly complex when DTs aggregate data across jurisdictions with different privacy regulations, medical device approval processes, and healthcare delivery frameworks, creating regulatory arbitrage opportunities and enforcement challenges that existing international law mechanisms struggle to address.

## Minor Social Themes

Environmental sustainability remained as a singular minor theme after our clustering. This theme’s relative neglect in our analysis may reflect our healthcare focus. If the analysis had instead examined DTs for the built environment or energy systems, for instance, environmental sustainability would presumably have achieved major status given the substantial computational demands and carbon footprint of large-scale infrastructure modelling systems.

Regardless, the environmental sustainability concerns surrounding DTs may prove increasingly significant as implementations scale. The computational demands for continuous modelling, real-time data processing, and comprehensive patient monitoring require substantial energy resources and cloud infrastructure that contribute to carbon emissions. The relative neglect of environmental considerations in current DT discourse mirrors broader patterns in healthcare technology adoption, where immediate clinical benefits often overshadow longer-term sustainability concerns, suggesting this minor theme may require proactive attention as healthcare systems increasingly recognise their environmental responsibilities.

## Minor Barrier Themes

The three minor barrier themes—regulatory and legal gaps, collaboration and engagement, and economic and commercial barriers—represent critical enablers requiring attention despite their classification as minor themes.

The *regulatory and legal gaps* theme reveals a critical disconnect where papers frequently cite regulatory uncertainty as an implementation barrier (see Table 1 in main manuscript), but the literature offers surprisingly little engagement with how adaptive regulatory frameworks might evolve alongside continuously learning DT systems^[[3]](#footnote-3)^. Current discussions centre on navigating existing approval pathways rather than reimagining regulatory paradigms for technologies that defy traditional medical device categories. Similarly, the *collaboration and engagement* theme identifies multidisciplinary requirements and stakeholder involvement as essential, yet the literature lacks concrete frameworks for sustaining such collaboration across professional silos, reconciling divergent disciplinary epistemologies, or managing the resource burdens of extended co-design processes. This latter concern is related, of course, to the *economic and commercial barriers* theme, which exposes perhaps the starkest gap—while studies acknowledge prohibitive infrastructure costs there is little in the way of alternative economic models, public-private partnerships, or mechanisms for preventing the capture of public health benefits by commercial interests.

# Methodological Limitations

Several limitations should be noted regarding our methodology:

- **Language bias**: All sources reviewed were written in English, which limits the generalisability of our thematic review and subsequent recommendations.
- **Selection bias**: Our choice of search engines for conducting the literature search will have created a selection bias towards more technical and practical papers (e.g. IEEE, PubMed). This is probably why we saw poor representation of the legal issues in particular. However, we tried to mitigate this through the augmentation stage (i.e. using ResearchRabbit) and also by adding a more general search engine (i.e. ScienceDirect).
- **Search Query Scope**: Related to the above limitation, our search scope will have also precluded papers that may have been pertinent to the ethical, legal, or social issues, but not directly concerned with digital twins. For instance, a paper that discussed legal issues in ‘modelling and simulation’ but did not mention ‘digital twins’ specifically would probably not have been picked up by our search query. Ultimately, this design choice will have resulted in missed sources, but also allowed us to be more focused and thorough with a smaller set of sources. That is, it is a representation of the “digital twinning” literature, on the basis that this is how the literature authors chose to present their research.
- **Subjective interpretation**: The coding process, while systematic, involved subjective interpretation of textual content, and the final thematic framework reflects the perspectives and disciplinary backgrounds of the coders involved. We tried to minimise this bias by having three coders involved in the process.
- **Evolving field**: The literature search was conducted at a specific point in time (2024), and the rapidly evolving nature of digital twin technologies means that new ELSI considerations may emerge that are not captured in this analysis. We have tried to mitigate this limitation, by identifying specific gaps and also making prescriptive recommendations that others can build upon.
- **Category imbalance**: The mixed-methods analysis revealed systematic differences in mean composite scores across categories (barriers: 0.528, ethical: 0.442, social: 0.302, legal: 0.279). Whilst our category-specific analysis addresses this, the global analysis may still undervalue social and legal themes due to their inherently lower frequency of discussion in the literature.
- **Co-occurrence interpretation**: The barrier-issue co-occurrence analysis demonstrates high connectivity (99.1% average) but did not seek to establish direct or causal relationships for the specific papers. That is, we did not determine whether any of the barriers were directly linked with specific ethical, legal, or social issues within a particular paper. While this may have proved interesting, we deemed the level of effort to be disproportionately high for the purpose of this review.
- **Geographic and regulatory bias**: Our findings predominantly reflect English-language discourse dominated by European and North American regulatory perspectives (GDPR, MHRA, EMA). Generalizability to healthcare systems in other regions with different regulatory frameworks, cultural values, and resource constraints remains uncertain. Digital twin implementations in low- and middle-income countries may face distinct ELSI challenges not captured in our analysis.
- **Anticipatory versus evidence-based findings**: The literature reviewed represents early-stage discourse about emerging technologies. Most concerns identified are anticipatory rather than evidence-based—for instance, discrimination concerns extrapolate from genetic testing precedents rather than documented DT-related harms. This review should be interpreted as mapping speculative risks and opportunities rather than confirmed issues from mature implementations.
- **Thematic analysis methodology**: While three independent coders participated, we did not quantify inter-rater reliability metrics (e.g., Cohen’s kappa). The consolidation from 182 to 34 codes involved subjective judgment about thematic equivalence. Our semi-systematic approach using ResearchRabbit for augmentation, while effective for discovery, introduces subjective judgment that may affect reproducibility.

Despite these limitations, our systematic approach provides a robust foundation for understanding the current state of ELSI considerations in digital twins for healthcare.

#

# References

Armeni, Patrizio, Patrizio Armeni, Irem Polat, et al. 2022. “Digital Twins in Healthcare: Is It the Beginning of a New Era of Evidence-Based Medicine? A Critical Review.” *Journal of Personalized Medicine*, 14. <https://doi.org/10.3390/jpm12081255>.

Ashraf, Taniya, Mohammad Ahsan Chisti, and Mohamed Mahees Raheem. 2024. “Digital Twin for Neurology: An Introduction to a New Frontier in Healthcare.” *2024 21st Learning and Technology Conference (L&T)*, 6. <https://doi.org/10.1109/LT60077.2024.10469438>.

Barricelli, Barbara Rita, Barbara Rita Barricelli, Elena Casiraghi, Elena Casiraghi, Daniela Fogli, and Daniela Fogli. 2019. “A Survey on Digital Twin : Definitions, Characteristics, Applications, and Design Implications.” *IEEE Access : Practical Innovations, Open Solutions*, 19. <https://doi.org/10.1109/access.2019.2953499>.

Beauchamp, Tom L, and James F Childress. 2013. *Principles of Biomedical Ethics*. 7th ed. Oxford University Press.

Begishev, Ildar, Albina Shutova, Elvir Akhmetshin, Veronika Denisovich, Elvira Latypova, and Ruslan Gilmanov. 2024. “Digital Twins in Healthcare System: Communication Between Society and Law.” *2024 Communication Strategies in Digital Society Seminar (ComSDS)*, 4. <https://doi.org/10.1109/ComSDS61892.2024.10502083>.

Braun, Matthias. 2021. “Represent Me: Please! Towards an Ethics of Digital Twins in Medicine.” *Journal of Medical Ethics*, 7. <https://doi.org/10.1136/medethics-2020-106134>.

Braun, Matthias, and Jenny Krutzinna. 2022. “Digital Twins and the Ethics of Health Decision-Making Concerning Children.” *Patterns* 3 (4): 7. <https://doi.org/10.1016/j.patter.2022.100469>.

Bruynseels, Koen, Filippo Santoni de Sio, and Jeroen van den Hoven. 2018. “Digital Twins in Health Care: Ethical Implications of an Emerging Engineering Paradigm.” *Frontiers in Genetics* (Switzerland) 9: 31. <https://doi.org/10.3389/fgene.2018.00031>.

Chang, Hung-Ching, Antony M. Gitau, Siri Kothapalli, Danny R. Welch, Mihaela E. Sardiu, and Matthew D. McCoy. 2023. “Understanding the Need for Digital Twins’ Data in Patient Advocacy and Forecasting Oncology.” *Frontiers in Artificial Intelligence* (Switzerland) 6: 5. <https://doi.org/10.3389/frai.2023.1260361>.

Chase, Geoffrey J., Cong Zhou, Jennifer L. Knopp, et al. 2023. “Digital Twins and Automation of Care in the Intensive Care Unit.” In *Cyber-Physical-Human Systems: Fundamentals and Applications*. <https://doi.org/10.1002/9781119857433.ch17>.

Coorey, Genevieve, Gemma A. Figtree, David F. Fletcher, et al. 2022. “The Health Digital Twin to Tackle Cardiovascular Disease-a Review of an Emerging Interdisciplinary Field.” *NPJ Digital Medicine* (England) 5 (1): 12. <https://doi.org/10.1038/s41746-022-00640-7>.

Croatti, Angelo, Angelo Croatti, Matteo Gabellini, et al. 2020. “On the Integration of Agents and Digital Twins in Healthcare.” *Journal of Medical Systems*, 8. <https://doi.org/10.1007/s10916-020-01623-5>.

Dahir, Hazim, Jeff Luna, Ahmed Khattab, Kaouther Abrougui, and Raj Kumar. 2023. “Chapter 4 - Challenges of Digital Twin in Healthcare.” In *Digital Twin for Healthcare*, edited by Abdulmotaleb El Saddik. Academic Press. <https://doi.org/10.1016/B978-0-32-399163-6.00009-3>.

Drummond, David, and Adrien Coulet. 2022. “Technical, Ethical, Legal, and Societal Challenges With Digital Twin Systems for the Management of Chronic Diseases in Children and Young People.” *Journal of Medical Internet Research* (Canada) 24 (10): 13. <https://doi.org/10.2196/39698>.

Fuller, Aidan, Zhong Fan, Charles Day, and Chris Barlow. 2020. “Digital Twin: Enabling Technologies, Challenges and Open Research.” *IEEE Access* 8 <https://doi.org/10.1109/ACCESS.2020.2998358>.

Huang, Pei-Hua, Ki-Hun Kim, and Maartje Schermer. 2022. “Ethical Issues of Digital Twins for Personalized Health Care Service: Preliminary Mapping Study.” *Journal of Medical Internet Research* (Canada) 24 (1): 12. <https://doi.org/10.2196/33081>.

Iqbal, Jeffrey David, Michael Krauthammer, and Nikola Biller-Andorno. 2022. “The Use and Ethics of Digital Twins in Medicine.” *The Journal of Law, Medicine & Ethics : A Journal of the American Society of Law, Medicine & Ethics* (England) 50 (3): 14. <https://doi.org/10.1017/jme.2022.97>.

Kamel Boulos, Maged N., and Peng Zhang. 2021. “Digital Twins: From Personalised Medicine to Precision Public Health.” *Journal of Personalized Medicine* (Switzerland) 11 (8): 12. <https://doi.org/10.3390/jpm11080745>.

Kerckhove, Derrick de. 2021. “The Personal Digital Twin, Ethical Considerations.” *Philosophical Transactions. Series A, Mathematical, Physical, and Engineering Sciences* (England) 379 (2207): 12. <https://doi.org/10.1098/rsta.2020.0367>.

Lauer-Schmaltz, Martin Wolfgang, Philip Cash, and David Gabriel Tacdeo Rivera. 2024. “ETHICA: Designing Human Digital Twins—a Systematic Review and Proposed Methodology.” *IEEE Access : Practical Innovations, Open Solutions* 12: 27. <https://doi.org/10.1109/ACCESS.2024.3416517>.

Leo, Carlo Giacomo, Maria Rosaria Tumolo, Saverio Sabina, et al. 2022. “Health Technology Assessment for In Silico Medicine: Social, Ethical and Legal Aspects.” *International Journal of Environmental Research and Public Health* (Switzerland) 19 (3): 13. <https://doi.org/10.3390/ijerph19031510>.

Li, Teng, Yi Shen, Yuxiang Li, Yongbo Zhang, and Song Wu. 2024. “The Status Quo and Future Prospects of Digital Twins for Healthcare.” *EngMedicine* 1 (3): 7. <https://doi.org/10.1016/j.engmed.2024.100042>.

Lupton, Deborah. 2021. “Language Matters: The ’Digital Twin’ Metaphor in Health and Medicine.” *Journal of Medical Ethics* (England), 1. <https://doi.org/10.1136/medethics-2021-107517>.

Mohr, Alex E., Carmen P. Ortega-Santos, Corrie M. Whisner, Judith Klein-Seetharaman, and Paniz Jasbi. 2024. “Navigating Challenges and Opportunities in Multi-Omics Integration for Personalized Healthcare.” *Biomedicines* (Switzerland) 12 (7): 23. <https://doi.org/10.3390/biomedicines12071496>.

Popa, Eugen Octav, Eugen Octav Popa, Mireille van Hilten, et al. 2021. “The Use of Digital Twins in Healthcare: Socio-Ethical Benefits and Socio-Ethical Risks.” *Life Sciences, Society and Policy*, 25. <https://doi.org/10.1186/s40504-021-00113-x>.

Sharma, Bhanu, Deepak Kaushal, Manu Sharma, Sudhanshu Joshi, Sanghmitra Gopal, and Priyanka Gupta. 2023. “Integration of AI, Digital Twin and Internet of Medical Things (IoMT) for Healthcare 5.0: A Bibliometric Analysis.” *2023 International Conference on Advances in Computation, Communication and Information Technology (ICAICCIT)*, 6. <https://doi.org/10.1109/ICAICCIT60255.2023.10466141>.

Shengli, Wei. 2021. “Is Human Digital Twin Possible?” *Computer Methods and Programs in Biomedicine Update* 1: 8. <https://doi.org/10.1016/j.cmpbup.2021.100014>.

Vallée, Alexandre. 2024. “Envisioning the Future of Personalized Medicine: Role and Realities of Digital Twins.” *Journal of Medical Internet Research* (Canada) 26: 13. <https://doi.org/10.2196/50204>.

Winter, Peter D., and Timothy J. A. Chico. 2023. “Using the Non-Adoption, Abandonment, Scale-Up, Spread, and Sustainability (NASSS) Framework to Identify Barriers and Facilitators for the Implementation of Digital Twins in Cardiovascular Medicine.” *Sensors (Basel, Switzerland)* (Switzerland) 30 (14): 30. <https://doi.org/10.3390/s23146333>.

Zhang, Kang, Hong-Yu Zhou, Daniel T. Baptista-Hon, et al. 2024. “Concepts and Applications of Digital Twins in Healthcare and Medicine.” *Patterns (New York, N.Y.)* (United States) 5 (8): 15. <https://doi.org/10.1016/j.patter.2024.101028>.

1. The frequencies shown here are deduplicated frequencies. That is, if a single paper had multiple annotations that were labelled with the same code, these were not double counted. However, we did use the raw frequencies in a subsequent analysis (see stage [2.6](#subsec:supp-stage6)) [↑](#footnote-ref-1)
2. The notably low threshold for social issues (0.147) reflected tight clustering of scores, with only one code being a clear outlier (environmental-sustainability at 0.051). [↑](#footnote-ref-2)
3. An exception being the recent proposal mentioned earlier by the FDA, MHRA, and Health Canada [↑](#footnote-ref-3)
